# Sex Distribution of Paper Mulberry (*Broussonetia papyrifera*) in the Pacific

**DOI:** 10.1371/journal.pone.0161148

**Published:** 2016-08-16

**Authors:** Johany Peñailillo, Gabriela Olivares, Ximena Moncada, Claudia Payacán, Chi-Shan Chang, Kuo-Fang Chung, Peter J. Matthews, Andrea Seelenfreund, Daniela Seelenfreund

**Affiliations:** 1 Departamento de Bioquímica y Biología Molecular, Facultad de Ciencias Químicas y Farmacéuticas, Universidad de Chile, Santiago, Chile; 2 Centro de Estudios Avanzados en Zonas Áridas (CEAZA), La Serena, Chile; 3 National Museum of Prehistory, Taitung 95060, Taiwan; 4 Biodiversity Research Center, Academia Sinica, Nangang, Taipei 11529, Taiwan; 5 National Museum of Ethnology, Osaka, Japan; 6 Escuela de Antropología, Universidad Academia de Humanismo Cristiano, Santiago, Chile; Washington University, UNITED STATES

## Abstract

**Background:**

Paper mulberry (*Broussonetia papyrifera* (L.) L'Hér. ex Vent) is a dioecious tree native to East Asia and mainland Southeast-Asia, introduced prehistorically to Polynesia as a source of bark fiber by Austronesian-speaking voyagers. In Oceania, trees are coppiced and harvested for production of bark-cloth, so flowering is generally unknown. A survey of botanical records of paper mulberry revealed a distributional disjunction: the tree is apparently absent in Borneo and the Philippines. A subsequent study of chloroplast haplotypes linked paper mulberry of Remote Oceania directly to a population in southern Taiwan, distinct from known populations in mainland Southeast-Asia.

**Methodology:**

We describe the optimization and use of a DNA marker designed to identify sex in paper mulberry. We used this marker to determine the sex distribution in selected localities across Asia, Near and Remote Oceania. We also characterized all samples using the ribosomal internal transcribed spacer sequence (ITS) in order to relate results to a previous survey of ITS diversity.

**Results:**

In Near and Remote Oceania, contemporary paper mulberry plants are all female with the exception of Hawaii, where plants of both sexes are found. In its natural range in Asia, male and female plants are found, as expected. Male plants in Hawaii display an East Asian ITS genotype, consistent with modern introduction, while females in Remote Oceania share a distinctive variant.

**Conclusions:**

Most paper mulberry plants now present in the Pacific appear to be descended from female clones introduced prehistorically. In Hawaii, the presence of male and female plants is thought to reflect a dual origin, one a prehistoric female introduction and the other a modern male introduction by Japanese/Chinese immigrants. If only female clones were dispersed from a source-region in Taiwan, this may explain the absence of botanical records and breeding populations in the Philippines and Borneo, and Remote Oceania.

## Introduction

Prehistoric settlement of the Pacific involved the intentional transport of many plant and animal species of economic value [1, 2, 3, 4, 5], and concluded with settlement of the islands of eastern Polynesia approximately 1000 years before present (BP) [6, 7, 8]. Plant species introduced into Oceania included food, medicinal and other economically important plants as part of a ‘transported landscape’ strategy for cultural reproduction in the newly settled islands [1, 2]. It has been estimated that around 70 plant species were dispersed by prehistoric Austronesian speaking voyagers into Polynesia [9]. Not all plants reached all islands, and only a small number of these reached islands as distant as Rapanui (Easter Island), Hawaii or New Zealand [4]. One of the plants introduced to Remote Oceania was paper mulberry, which appears to be native to mainland and subtropical Southeast and East Asia, as far east as Taiwan [3; 10, 11]. In Remote Oceania, paper mulberry is well known as the main source of the highest quality fiber for making bark-cloth for mats, clothing and cordage for practical and ceremonial or ritual purposes. The tree is one of the few prehistoric introductions into Remote Oceania that is not a food plant.

### Paper mulberry dispersal

The plant family to which paper mulberry belongs, the Moraceae, includes figs and many other plants that provide food and fiber [5, 12]. A northern sister-species, *B*. *kazinoki*, is the main source of fiber for handmade paper in Japan, where *B*. *papyrifera* is known as an introduced species [3]. Paper mulberry is a fast growing shrub or tree, diploid (2*n* = 26), and dioecious (with male and female flowers on separate individuals). The male inflorescence is a dense, many-flowered, pendulous catkin of 4–8 cm; the female inflorescence is a dense, many-flowered, globose head of about 2 cm, and the fruit is a collection of a small fleshy orange to red druplets [13]. The sweet druplets are highly attractive to birds. When both male and female plants are introduced to the same area, wind pollination makes it relatively easy for breeding populations to become established. Such establishment has been observed in diverse environmental conditions, in the Phillipines, Pakistan, Japan, and the Solomon Islands. From herbarium records and floristic accounts, Matthews [3] mapped the distribution of paper mulberry in Asia and the Pacific, and found that males, females and breeding populations were present in East and Southeast Asia. The tree was apparently absent in the Philippines, Borneo and Micronesia, while present in Indonesia, Melanesia and Polynesia. Not including known modern introductions, the sex of trees introduced to Indonesia, Melanesia and Polynesia was unknown. The apparent disjunction or bottleneck in island Southeast Asia was unexpected, has been confirmed in the process of collecting samples for later surveys of genetic variation in paper mulberry, and remains unexplained

In a survey of *B*. *papyrifera* samples from Polynesia, Southeast Asia and East Asia, we analysed non-coding internal transcribed spacer (ITS) sequences of nuclear ribosomal DNA, and inter-simple sequence repeat (ISSR) sequences in total DNA extracts. Diversity was found in Asia and not in Polynesia [14], with the sole exception of Hawaii [15]. The data suggested a prehistoric human-mediated movement of paper mulberry from East Asia to Polynesia, by an unknown route through island Southeast Asia, and a second, possibly historic, human-mediated introduction to Hawaii [15]. A subsequent study of chloroplast haplotypes in paper mulberry by Chang et al. [11] indicated that the most common variant of paper mulberry found from Indonesia to Melanesia and Polynesia, and the one most likely introduced by the early colonists in Polynesia, has an origin in southern Taiwan. The fact that a single haplotype, cp-17, is dominant across this vast region is consistent with the dispersal of plants of a single sex to Remote Oceania, but does not prove this.

In Oceania, most traditional crops, except for coconuts and a few others, are propagated asexually [16, 17, 18, 19]. Very few dioecious plants were introduced prehistorically into Remote Oceania such as the narcotic plant kava (*Piper methystichum*) a well-studied dioecious domesticate that was introduced from Vanuatu into Polynesia [16], and the greater yam (*Dioscorea alata*) [20]. Sexual reproduction of these species is dependent on the presence of both mature female and male plants in some proximity, for pollination and seed production. Paper mulberry naturally suckers from the root system, both in the wild and in cultivation, and is easily propagated by the use of cuttings [3]. In the absence of pollination and seed production, long-distance dispersal must depend entirely on humans. In Remote Oceania, paper mulberry stems are usually cut approximately every two years when they reach about 2 to 2.5 meters height for harvesting the bark to obtain fibers, so the plants do not reach maturity, and rarely develop flowers [21]. As a result, the sex of paper mulberry plants is usually not seen, and when botanical specimens are collected for herbaria, floral parts are generally lacking [3].

**Fig 1:**
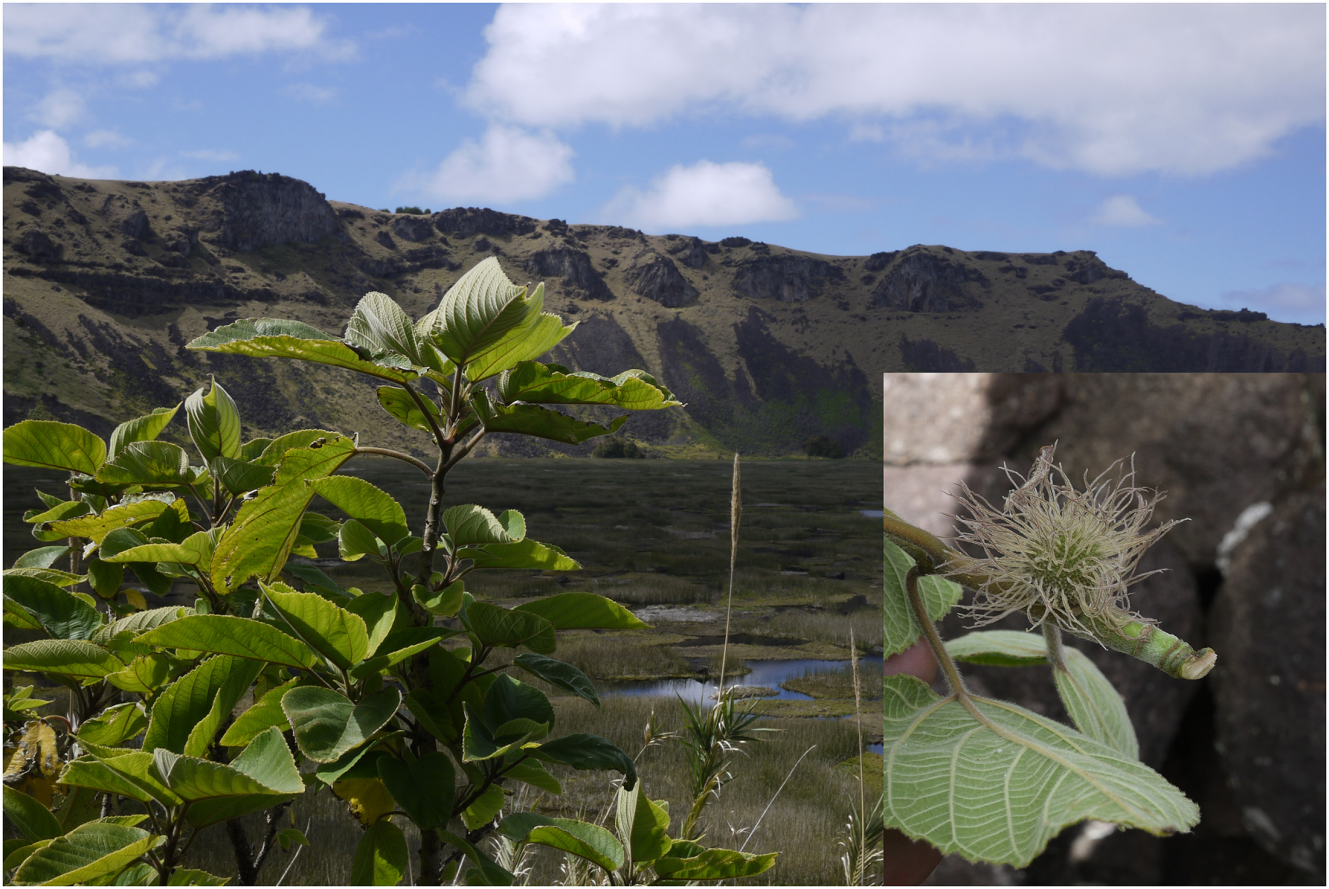
Female flowering *of B*. *papyrifera*, Rapanui, 2014. Tree at left, small branch with female inflorescence at right. The stigmas are exerted but no male tree is nearby for wind pollination. This crater population is isolated and not frequently harvested.

A relict population of paper mulberry has long been known to exist in a crater on Rapanui [22, 23], and was found to produce female flowers (Fig 1), but the sex of paper mulberry in Remote Oceania generally remained unknown.

### The question of sex of paper mulberry in the Pacific

Matthews [3] noted that the population of paper mulberry plants now present in Polynesia could be descended from one individual of either sex brought by the original human colonizers or from individuals of both sexes which may or may not have been able to reach the same islands and form breeding populations. Whistler and Elevitch [9] asserted that all specimens in Polynesia might be/are male clones, but we have not found evidence to support this, in either botanical or palaeobotanical reports. One reason to suggest that only one sex of paper mulberry was introduced into the Pacific is its non-invasive nature there. When both sexes are introduced into non-native areas, paper mulberry quickly disrupts the native habitat, becoming highly invasive, choking out native flora in its competition for nutrients, space and light [3, 24, 25, 26].

Male *B*. *papyrifera* plants produce large quantities of pollen, as attested in India and Pakistan [27; 28, 29] where the airborne pollen of large numbers of male plants have become a public health problem. Since paper mulberry in Polynesia generally is cut before it flowers, it seems unlikely that pollen or seeds will be found in archaeological sites [3]. No large amounts of pollen belonging to Moraceae have ever been identified in archaeological sites in Polynesia, and those found are likely to represent native Moraceae and/or Urticaceae species (see also [30]). Pollen types are not very distinct among different species and genera of Moraceae, making the identification of *Broussonetia* species difficult. The absence of verified paper mulberry pollen in palaeoecological contexts does not disprove the past presence of male plants, or prove the presence of only female plants in an area where the tree is present today.

We are interested in the sex distribution of paper mulberry in Oceania because the question of sex has direct bearing on whether or not the plant can naturalize, form breeding populations, and survive without human assistance.

In dioecious plants sex determination genes and pathways are not universal. No master sex determination gene has been identified, and many genes that affect sex determination have been found [31]. In some plants, the development into female or male is controlled by epigenetic signals or diverse environmental stimuli [31]. Very little is known at the molecular level of sex-specific DNA sequences and the mechanisms of sex differentiation in plants. Recently Wang et al. [32] identified a molecular sex marker for *B*. *papyrifera*. A comparison of gene sequences for *Eucommia ulmoides* Oliv. [33], *Trichosanthes dioica* [34], *Carica papaya* [35], *Pistacia vera* [36] and paper mulberry [32] does not show common sex-defining sequences in these different species and genera.

In the present paper, we have identified the sex of paper mulberry in Near and Remote Oceania in order to determine if one or both sexes are present, and to reconsider the possible mechanisms of dispersal and survival of the tree in the light of this information.

In work aimed at helping plant breeders create new cultivars, Wang et al. [30] identified a short male-specific DNA sequence in *B*. *papyrifera*. To distinguish the sex of these plants more effectively, we developed a duplex PCR protocol based on the male-specific sequence recorded in GenBank (HQ202152.1) [32]. We then characterized the sex distribution of living plants on 25 islands in 13 archipelagos or island groups across a broad region of Near and Remote Oceania. We also located a small number of flowering paper mulberry specimens in herbarium collections from the Pacific. For the purposes of interpretation, we compare the geographical sex distribution with the distributions of nuclear ribosomal DNA ITS-1 variants and chloroplast haplotypes. In our sample set, female trees predominate across the introduced range from island Southeast Asia to Polynesia. Exceptions are few and are thought to reflect known or likely modern introduction. The results allow us to solve the long-standing question of the sex distribution of this dioecious species in Remote Oceania and suggest a new explanation for the absence of botanical records of paper mulberry in Borneo and the Philippines, reconfirm the dependence of this crop on human transmission outside the natural range, and point to a strategy for avoiding the spread of invasive breeding populations outside the natural range.

## Materials and Methods

The Corporación Nacional Forestal (CONAF) from Chile issued a research permit for collecting inside the National Park on Easter Island. The Département de la Récherche, French Polynesia issued permits for collecting in the Marquesas, Tahiti and Austral Islands; the Province Museum of Central Sulawesi aided in sampling in Sulawesi, Indonesia. The Herbarium Pacificum (B. P. Bishop Museum, Hawaii), the Auckland Herbarium (Auckland Institute and Museum, New Zealand) and the US National Herbarium (Smithsonian Institute, USA) issued permits for herbarium sampling.

### Archival research

Plant specimens were examined directly or online at the following locations: (1) B.P. Bishop Museum, Honolulu, USA; (2) Herbarium Bogoriense, Bogor, Indonesia; (3) National Museum of Natural History, Santiago, Chile; (4) Auckland Herbarium, Auckland Institute and War Memorial Museum, New Zealand, (5) Landcare NZ, Lincoln, New Zealand, (6) National Herbarium, Smithsonian Institution, Washington D.C., USA., and (7) National Museum of Natural History, Paris, France.

### Field areas, sampling of plant material and DNA extractions

Young leaves were collected in New Caledonia, Western Polynesia (Fiji, Samoa, Wallis and Tonga) and Eastern Polynesia (Marquesas archipelago [Ua Pou, Nuku Hiva, Fatu Hiva, Tahuata], Society Islands [Raiatea, Tahiti], Hawaii [Oahu, Big Island], Austral islands [Rapa], Pitcairn and Rapanui) between 2008 and 2014. Leaf samples collected in 2008 in Fiji, Samoa, Tonga, Marquesas, Tahiti, Pitcairn, Raiatea and Rapanui were preserved at -20°C in the laboratory [10]. Leaf samples collected later in Sulawesi, New Caledonia, Wallis, Fiji, Tonga, Hawaii, Rapanui, Rapa and Marquesas were dried with silica gel and stored at room temperature. Leaf samples collected in Taiwan, China, Japan and Vietnam were dried in silica gel. Samples from Rapa and New Caledonia were kindly provided by J.-Y. Meyer and A. Brianchon, respectively and samples from Pitcairn were collected by C. Walter. Additionally five herbarium samples from New Guinea and the Solomon islands were included in the study.

The origin of all 340 contemporary and the 12 herbarium samples are shown in Table 1, and include 33 samples from Asia, 26 (18 contemporary and eight herbarium) samples from Near Oceania and 293 (289 contemporary and four herbarium)samples from Remote Oceania, as shown in S1 and S2 Tables. Sampling was not evenly distributed, because the abundance of plants on each island or island group is highly variable, as described for Remote Oceania in Seelenfreund et al. [10]. Herbarium samples were provided by the Auckland Herbarium (Accessions AKA 116673 from New Guinea; AKA 214298 from the Solomon Islands), and the B.P. Bishop Museum Pacific Herbarium (BISH 161323 from New Guinea; BISH 416666 and BISH 505999 from the Solomon Islands, BISH 161324, BISH 161326, BISH 32928, BISH 757984 from Fiji).

**Table 1:** List of samples analyzed.

	Location	number of samples	ITS-1	Sex marker
Country	Archipelago/			
	Island/			
	Region			
Indonesia	Sulawesi	16	G	Female
New Guinea*	New Guinea	5	G	Female
Solomon Islands*	Guadalcanal	5	G	Female, Male
New Caledonia	Grande Terre	3	T	Female
Fiji *	Viti Levu	9	T	Female
	Taveuni	21	T	Female
	Vanua Levu	28	T	Female
	Vatulele	19	T	Female
	Namuka i Lau	1	T	Female
	Koro	1	T	Female
Tonga	Tongatapu	36	T	Female
	Vava'u	6	T	Female
	Eua	10	T	Female
Samoa	Upolu	5	T	Female
	Savaii	14	T	Female
Frenchoverseas territory	Wallis	12	T	Female
French Polynesia	Tahiti	6	T	Female
	Raiatea	1	T	Female
	Marquesas- Ua Pou	2	T	Female
	Marquesas-Nuku Hiva	2	T	Female
	Marquesas-Hiva Oa	1	T	Female
	Marquesas-Tahuata	3	T	Female
	Marquesas-Fatu Hiva	5	T	Female
	Austral Islands (Rapa)	2	T	Female
British overseas territory	Pitcairn	6	T	Female
Chile	Rapanui	62	T	Female
USA	Hawai- Big Island	21	T, G	Female, Male
	Hawai- Oahu	17	T, G	Female, Male
Taiwan	Taiwan	19	G	Female, Male
Vietnam	North Vietnam	5	G	Female, Male
Japan	Honshu	5	G	Female, Male
China	Guangdong Province	4	G	Female, Male

*Indicates herbarium samples (New Guinea: 5 samples, Salomon Islands: 5 samples, Fiji: 4 samples; for details see S2 Table)

Four samples collected in Taiwan were used as sex controls, and came from two male plants (BQUCH0137, BQUCH0138) and two female plants (BQUCH0139, BQUCH0140). The sex of these plants was determined in the field by visual observation of male and female inflorescences during the flowering season.

Genomic DNA was extracted following a CTAB extraction protocol described by Lodhi et al. [37] and modified as described in Moncada et al. [38]. Concentration of DNA was determined by fluorescence detection using the Quant-iT^™^ PicoGreen^®^ dsDNA Assay Kit (#P7589) as indicated by the supplier. Extracted DNA was stored in double distilled sterile water at -20°C until analysis.

### ITS-1 amplification and data analysis

The ITS-1 region was amplified using ITS-A (5´- GGA AGG AGA AGT CGT AAC AAG G -3`) and ITS-C (5´- GCA ATT CAC ACC AAG TAT CGC -3`) primers as described by Blattner [39]. Amplification followed the PCR protocol of Seelenfreund et al. [14]. All PCR reactions were set up in a UV-treated cabinet. Sequences were aligned using the MUSCLE algorithm and CLC Sequence Viewer 7.5 software [40].

### Primer design and duplex PCR for sex determination

We designed three primers based on the male-specific sequence of the *B*. *papyrifera* AFLP fragment isolated by Wang et al. [32], deposited under GenBank accession number HQ202152.1. The three primers were used to develop a duplex PCR protocol (see below), consisting of one forward primer MMF (5´- AGC CCT TTG GAT CGC GAC TTA GAA -3`) and two reverse primers, MMRS18 (5´- GCC ATG TCA ACG TCA TCA -3`) and MMRL (5´- CTG GAC AAG ACC AAC TTT GAA TCC G -3`). One of the reverse primers targets the male-specific sequence identified by Wang et al. [32], while the other targets a nearby sequence shared by both male and female plants. The primers designed by us and by Wang et al. [32] are shown in S1 Fig. The two amplicons produced with our primers differ in length by 147 base pairs, thus providing a binary sex marker that is hereafter referred to as the ‘Male Marker’. The duplex PCR reaction mixture consisted of 1.2 ng of DNA, 2.5 mM MgCl_2_, 0.625 mM of dNTPs, 5.5 pmol of the forward MMF primer, 4 pmol of the reverse MMRS18 primer and 1.5 pmol of the second reverse MMRL primer, 1 U of GoTaq^™^ Flexi DNA Polymerase and 1x PCR buffer (Promega, Madison, WI, USA) in a final volume of 20 μl.

The amplification program used an initial denaturation at 94°C for five min, and 30 cycles with a denaturation step at 94°C for one min, an annealing stage at 55°C for one min, and an elongation step at 72°C for 45 sec, followed by a final extension at 72°C for seven min. For each PCR assay, positive sex-control samples (DNA from one male and one female specimen) and a negative control (water) were included. All PCR reactions were set up in a UV-treated PCR cabinet.

### Scoring of the binary sex marker

**Fig 2:**
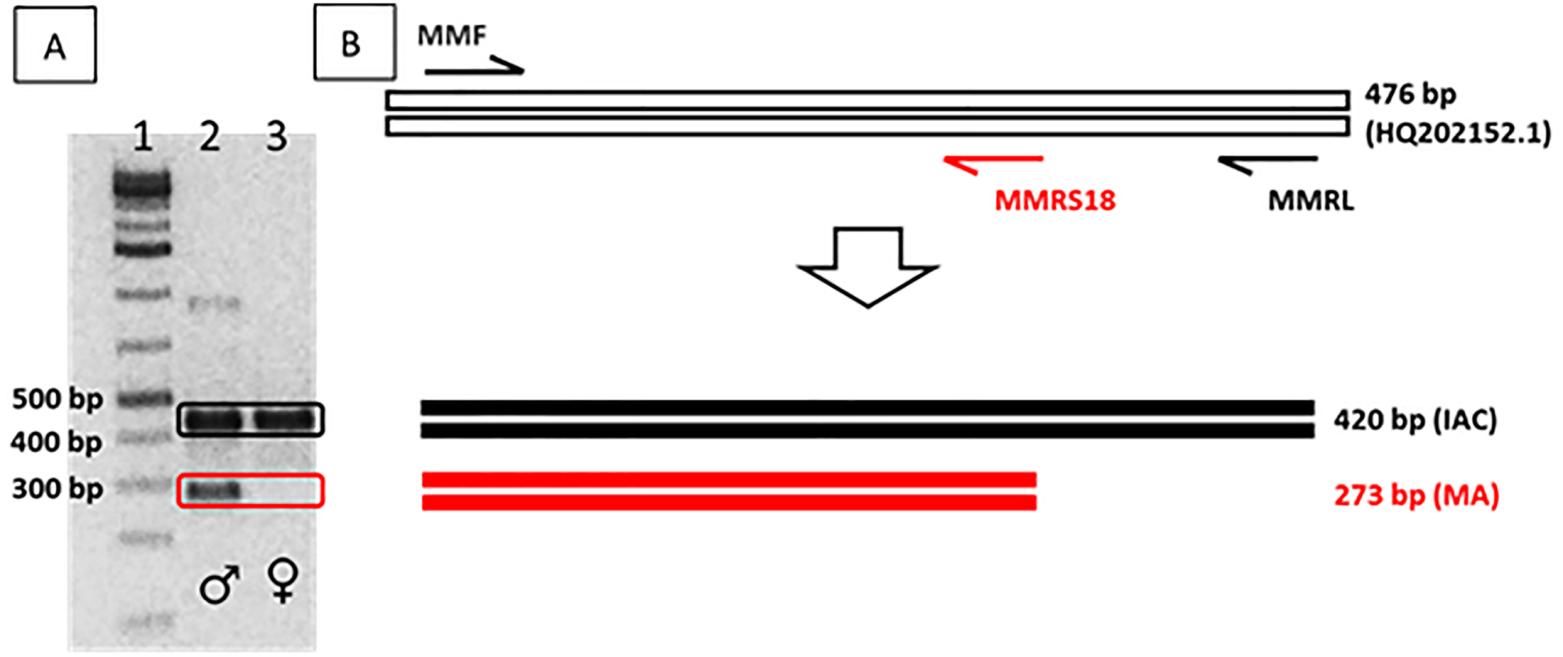
Diagram of duplex PCR for sex identification of *B*. *papyrifera*. A. Banding pattern of duplex PCR. Lane1: 1 kb Plus DNA Ladder, Lane 2: Male Amplification Pattern, Lane 3: Female Amplification Pattern. Electrophoresis on 1.5% agarose gel. B. Diagram of amplification products. IAC = Internal Amplification Control, MA = Male Amplification.

Amplicons were analyzed by electrophoresis on 1.5% agarose gels in 0.5 X TBE buffer, dyed with GelRed^™^ and visualized under UV light. A 1 kb plus DNA ladder (Thermo Scientific GeneRuler # SM1331) was included in every gel. Bands were photographed with a UV transilluminator and gel images were analyzed by visual inspection. All samples were analyzed in duplicate. Male individuals were identified by the presence of both the 273 and 420 bp bands. Female individuals displayed a single 420 bp band (Fig 2). Positive controls of male and female specimens were included for comparison in all assays.

### Sequencing and alignment of the sex-specific region

Amplicons were purified using the DNA Clean-up & Concentration purification kit from Zymo Research Corporation (Irvine, Ca., U.S.A.) according to the manufacturer’s instructions and sequenced by Macrogen Inc. (Seoul, South Korea). For comparison, sequences were aligned using the MUSCLE algorithm implemented in MEGA6 [41].

## Results

### Archival research

Of the seven herbaria examined (234 accessions) only 26 exhibited reproductive organs, and one had the sex indicated as part of the label (Table 1). Most paper mulberry specimens observed did not display flowers or fruits. A few from Rapa Nui, Hawaii and Pitcairn displayed only female flowers. The oldest collected specimens with female flowers are the specimens collected in Tahiti by Moerenhout in 1834. One specimen from Hawaii found at the B.P. Bishop Museum in Honolulu is a putative male plant (Table 2).

**Table 2:** List of herbarium accessions with inflorescences.

Herbarium	Island	Accession number	Year collected	Inflorescence
National Museum of Natural History; Paris, France	Tahiti	6758369	1834	female
National Museum of Natural History; Paris, France	Tahiti	6758371	1834	female
Auckland Institute & Museum; New Zealand	Solomon Islands (Guadalcanal)	214298	1903	female
National Herbarium, Smithsonian Institution; USA	Hawai (Big Island)	3160656	1909	female
National Herbarium, Smithsonian Institution; USA	Hawai (Big Island)	3160655	1910	female
B.P.Bishop Museum, Hawaii; USA	Hawai (Big Island)	58346	1909	female
National Museum of Natural History; Santiago, Chile	Rapanui	58271	1911	female
National Museum of Natural History; Santiago, Chile	Rapanui	58300	1911	female
B.P. Bishop Museum, Hawaii; USA	Hawai (Niihau)	58375	1912	female
B.P. Bishop Museum, Hawaii; USA	Hawai (Lanai)	58345	1914	female
B.P. Bishop Museum, Hawaii; USA	Hawai (Lanai)	58364	1914	female
National Herbarium, Smithsonian Institution; USA	Rapanui	1093802	1917	female
B.P. Bishop Museum, Hawaii; USA	Rapanui	161285	1917	female
B.P. Bishop Museum, Hawaii; USA	Hawai (Oahu)	58368	1918	female
B.P. Bishop Museum, Hawaii; USA	Rapa	161293	1921	female
B.P. Bishop Museum, Hawaii; USA	Hawai (Big Island)	58357	1924	female and male (*)
B.P. Bishop Museum, Hawaii; USA	Hawai (Molokai)	58352	1928	female
B.P. Bishop Museum, Hawaii; USA	Hawai (Oahu)	58387	1930	female
B.P. Bishop Museum, Hawaii; USA	Hawai (Big Island)	58347	1945	female
National Museum of Natural History; Santiago, Chile	Rapanui	75595	1953	female
National Museum of Natural History; Santiago, Chile	Rapanui	75595	1953	female
B.P. Bishop Museum, Hawaii; USA	Pitcairn	664608	1957	female
B.P. Bishop Museum, Hawaii; USA	Easter Island	129525	1971	female
B.P. Bishop Museum, Hawaii; USA	Solomon Islands (Guadalcanal)	416666	1977	female
B.P. Bishop Museum, Hawaii; USA	Solomon Islands (Guadalcanal)	505999	1985	female
National Museum of Natural History; Santiago, Chile	Rapanui	162504	2012	female

(*) This accession has no inflorescences; however, it was included in the list as it has two leaves each identified as coming from a plant of a different sex. In total, we checked 234 accessions of seven herbaria.

### Ribosomal DNA ITS-1 sequence analysis

The ITS-1 sequences displayed a G/T transversion at nucleotide position 203, as noted previously [14]. The G variant was found in all samples from China, Vietnam, Japan, Taiwan and 18 samples from Hawaii (Table 1). All Polynesian samples, excluding these 18 Hawaiian specimens, displayed the T variant at the indicated position (n = 269).

### Using duplex PCR to identify sex

The duplex PCR method reported here facilitated visual scoring of PCR products and unambiguous identification of male and female paper mulberry plants. Fig 2 shows a schematic representation of the male-specific region and the three primers designed for the duplex PCR, as described in Materials and Methods. The MMF-MMRL primer pair amplifies the 420 bp fragment present in both male and female individuals (Fig 2A, lanes 2 and 3, see also diagram in Fig 2B), congruent with the amplification controls. The MMF-MMRS18 primer pair generates an additional amplicon of 273 bp exclusively in male plants (compare lanes 2 and 3 in Fig 2A). The use of an amplification control in the duplex PCR allows the unambiguous sex identification of each individual, generating a differential and specific banding pattern for each sex.

**Fig 3:**
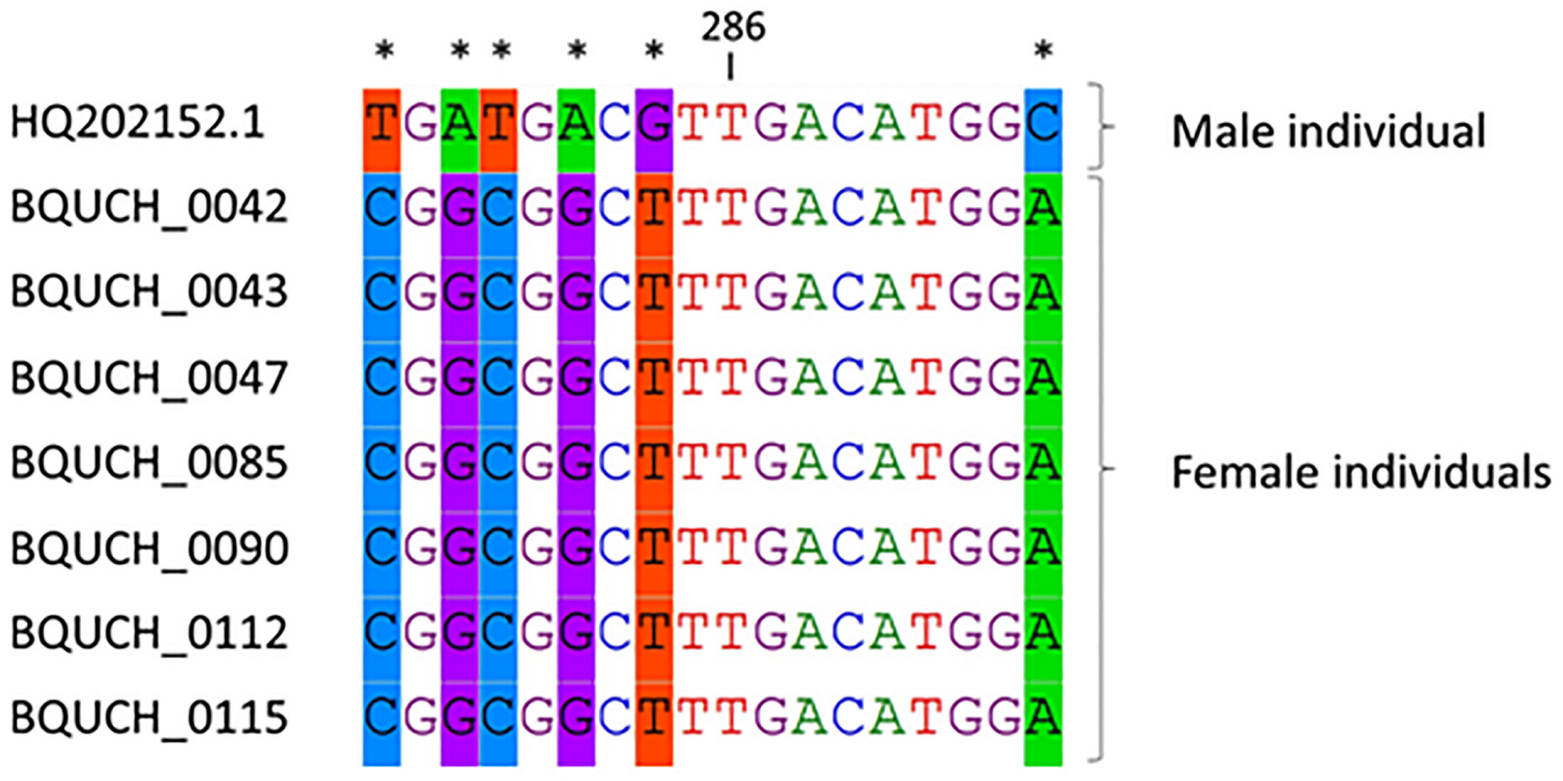
Alignment of male and female sequences in the polymorphic region of the *B*. *papyrifera* sex marker.

**Fig 4:**
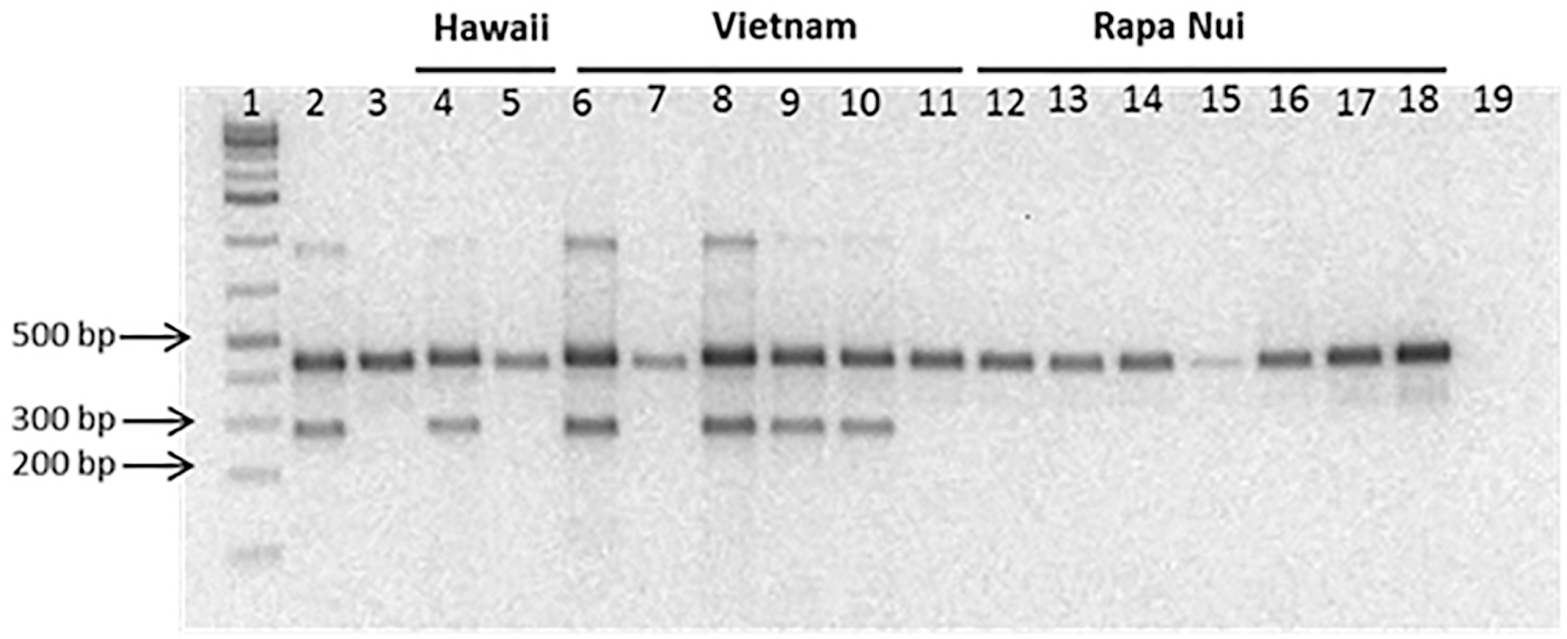
Banding pattern of a duplex PCR used to identify sex in selected *B*. *papyrifera* samples. Lane 1: 1Kb DNA standard, Lane 2: BQUCH0137 (male control); lane 3: BQUCH0139 (female control); lane 4: BQUCH0194; lane 5: BQUCH0195; lane 6: BQUCH0201; lane 7: BQUCH0202; lane 8: BQUCH0203; lane 9: BQUCH0204; lane 10: BQUCH0204d; lane 11: BQUCH0205; lane 12: BQUCH0208; lane 13: BQUCH0208d; lane 14: BQUCH0209; lane 15: BQUCH0210; lane 16: BQUCH02011; lane 17: BQUCH0212; lane 18: BQUCH013; lane 19: negative PCR control (H_2_O). Electrophoresis on 1.5% agarose gel.

The differential amplification of DNA from male and female plants with the MMF-MMRS18 primer pair is due to a six bp polymorphic region between the relative positions 277 to 294 within the HQ202152.1 sequence (Fig 3). The primer MMRS18 was designed to target these six polymorphic bases located within this small region, and to anneal only to the male-specific sequence. The sequences of one male individual (HQ202152.1.) and seven female individuals from different locations in Polynesia (Table 2 and S1 Table) are compared in Fig 3. Plants from the native range in Asia and from non-native range of the Pacific islands were typed using this Male Marker. As shown in Fig 4, the sex of each sample can be easily identified by visual inspection. The first two lanes correspond to male and female control samples, respectively. This assay is highly reproducible as exemplified by the analysis of biological replicates shown in lanes 9 and 10 (male plants) and lanes 12 and 13 (female plants), respectively. In addition, the replicate analysis of all samples consistently identified the same sex for each specimen.

### Sex distribution in Asia, Near and Remote Oceania

**Fig 5:**
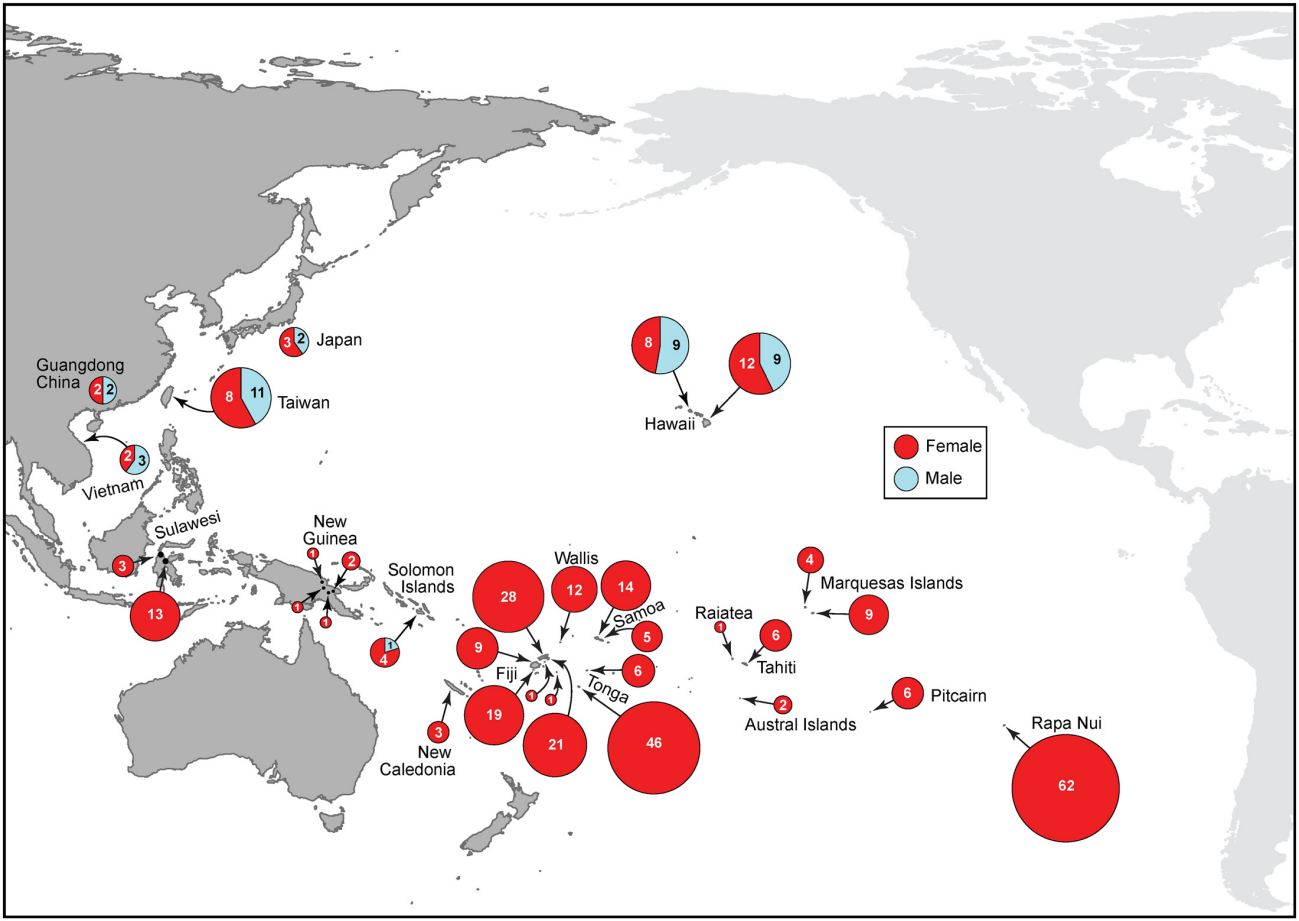
Sex distribution of paper mulberry in the sampled native range, Near and Remote Oceania. Size of circles is proportional to the number of samples (indicated inside each circle).

Analysis of 33 samples from the native range (China, Vietnam, Japan and Taiwan) indicated the presence of both male and female plants. The sex ratio was approximately even for all sampled locations, consistent with the known presence of breeding populations in eastern Asia [3]. Among paper mulberry plants introduced into Near and Remote Oceania only females were found, with the sole exception of Hawaii and two samples in the Solomon Islands, where both sexes are present (Fig 5). A total of 300 female plants were identified in Sulawesi, New Guinea, Solomon Islands, Fiji, Wallis, New Caledonia, Tonga, Samoa, Raiatea, Tahiti, Rapa, Marquesas, Pitcairn and Rapanui. The only locations in Oceania with male plants were the Solomon Islands and Hawaii. Among the Solomon Islands samples, four individuals were female, and only one specimen was a male plant. Among the 38 samples collected in Hawaii, we found 20 female plants (53%) and 18 male plants (47%). Inside Hawaii, our Big Island set (n = 21) included 12 females and nine males, and the Oahu Island set (n = 17) included eight females and nine males.

These results answer the long-standing question regarding the presence of a single sex of paper mulberry plants in Remote Oceania, revealing that the overwhelming majority of specimens are female plants. The geographical sex distribution of *B*. *papyrifera* found in the Pacific according to our results is depicted in Fig 5.

## Discussion

Because paper mulberry is dioecious, the spatial distribution of male and female plants is critical for sexual reproduction of the plant, and has implications for the formation of breeding populations, and for genetic diversity present in the introduced range of this species. Although today there is a growing tendency to use improvements in sequencing technologies and massive genetic analysis [3], this work is focused on the analysis of a single, binary marker (female/male) that provides relevant information using a simple molecular genetics analysis. The duplex PCR method reported here allows the sex of paper mulberry individuals to be identified unambiguously at any stage of growth (before or after flowering), is easily applied, and is affordable for large sample sets and population studies. In the future, it may be useful for plant breeders working with paper mulberry, and for ecological studies of both natural and naturalized populations.

In a former study, using chloroplast DNA sequences, we demonstrated a tight genealogical link between paper mulberry populations in South Taiwan and Remote Oceania by way of Sulawesi and New Guinea, presenting the first study, to our knowledge, of a commensal plant species transported to Polynesia whose phylogeographic structure concurs with expectations of the “out of Taiwan” hypothesis of Austronesian expansion [11]. Nonetheless, this study does not explain the sex distribution of this dioecious plant and its relationship to its dispersal history in the introduced range of Near and Remote Oceania, which corresponds to the aim of the present research.

On a wide geographical scale, the co-occurrence of male and female plants is an indication of where breeding populations might exist and provides a rough outline of the likely natural range [3]. Our results indicate that in locations within the likely natural range, from mainland Asia to Taiwan, both sexes are present at a similar ratio. The presence of male and female plants in close proximity permits pollination of female flowers, seed development and seed dispersal. Individual female trees can produce hundreds of fruit and thousands of seeds, and readily colonize open habitats nearby, as observed by us in Taiwan generally, and in the cities of Kyoto and Tokyo, where the tree has naturalized in some city districts after introduction.

The results of the archival research showed that the vast majority of plant materials kept in museum herbaria did not have reproductive organs that are usually included for taxonomic use. In the case of *B*. *papyrifera*, most samples from the Pacific do not bear fruits or flowers. To our knowledge, the first collectors in the Pacific were P. Commerson, the naturalist from L.A. de Bougainville’s expedition to the Pacific in 1768, and J. Banks and D. Solander, the botanists from J. Cook’s first expedition into the Pacific in 1769. These early collections testify to the plants present at a time before modern translocations and plant introductions to these islands [42]. Within the Banks and Solander collection we find specimens of *B*. *papyrifera*, labeled as coming from the Friendly Islands. As discussed previously [10] these are most probably from Tahiti and not from Tonga (Friendly Islands) since Cook did not visit these islands until much later. These specimens housed today at the Smithsonian Institute and Museum lack flowers, confirming that the plants they saw were regularly pruned and probably did not develop flowers. In contrast, the specimens collected by Moerenhout in Tahiti in 1834, were possibly taken from already partly abandoned stands, after the introduction of western clothing and the decline of tapa making. The consequent abandonment of plantations allowed plants to reach maturity and develop flowers. In the case of the Easter Island, specimens with female flowers have been collected since the early XX century from old plantations growing inside Rano Kao crater. The few flowering plants found on Easter Island today are old plants, and the feral remnants of old plantations.

On almost all islands of Near and Remote Oceania represented in our sample set, we found only female individuals among extant plants. Apart from the exception discussed below, the predominance of female individuals in this region is consistent with one of the scenarios suggested by Matthews [3], namely introduction and vegetative propagation of a single male or female clone. Our results contradict the suggestion by Whistler & Elevitch [9] that only male clones of paper mulberry are present in the Pacific (an inference made from the observation of male flowering in Hawaii; Whistler pers. comm. 2016). The results do not imply that all plants reaching a particular island came with the first human immigrants, or were introduced only once, and neither can we ascertain that only a single female clone was introduced. At first settlement and/or later, one or more female clones could have been introduced as elements of exchange networks that developed as island groups were settled and became more widely known.

In the case of the Solomon Islands, both the contemporary and herbarium samples that are all less than 50 years old were collected near Honiara, on Guadalcanal Island. As demonstrated in Chang et al. [11] all these plants are of recent introduction and are invasive, denoting that presence of both sexes. In addition, the presence of the G polymorphism in their ribosomal ITS1 sequence reinforces their Asian origin.

The presence of both female and male plants in Hawaii is probably the result of at least two introductions made during different historical periods, before and after modern colonization of the islands by immigrants from across Eurasia. The ITS-1 sequence displayed by all female plants in our Hawaiian sample set exhibits the same “T” polymorphism found in all other female plants in Remote Oceania. Strikingly, the male plants from Hawaii all display the “G” polymorphism found in Asian paper mulberry plants [14, 15]. Indeed, the male plants in the present study correspond to the same plants that displayed an Inter Simple Sequence Repeats (ISSR) genotype that is different from the genotype common across Polynesia [15]. The cumulative results strongly suggest that male and female plants in Hawaii represent separate introductions from different geographical sources, and that the females present the same genetic make-up as those found throughout Remote Oceania. The general overlap of our Polynesian sample set with the Polynesian sample set of Chang et al. [11], and specific shared samples used in both data sets, allow us to identify the chloroplast haplotype of the female samples as cp17. Interestingly, the widespread chloroplast haplotype cp 17 links the female paper mulberry populations from Hawaii and Remote Oceania to those of southern Taiwan [11], whereas the male plants from Hawaii present the East Asian chloroplast haplotype (cp-20), that is known only in China, or cp-41, a haplotype derived from cp-28 found in China and Japan, that is exclusive to Hawaii [11]. This is consistent with an introduction of male plants brought by Chinese and/or Japanese immigrants in the 19^th^ and 20^th^ centuries [11].

Why were male and female clones introduced to Hawaii, at different times by different people? One possibility is that there was a cultural or practical bias in the choice of paper mulberry plants taken to Remote Oceania by Austronesian voyagers and in the choice by Chinese or Japanese immigrants in historic times. In principle, male and female clones of paper mulberry may have certain agronomic or use traits preferred by different people at different times and places. The qualities required as a source of medicine and/or papermaking in East Asia, for example, may be unlike those required for bark-cloth manufacture. However, there is no relevant information on such differences and preferences in the regions of Southeast and East Asia where paper mulberry is still economically important. The past selection of female or male clones for propagation, in the source regions, could also have been incidental and unrelated to the sex of the plants. The periodical harvesting of clones in the introduced range, and concomitant absence of sexually mature plants, could have led to the unintentional loss of male plants, particularly if they were only rarely introduced. In the absence of breeding, outside the natural range of the species, new male and female offspring could not be generated and no further sex selection could be made.

In Hawaii today, the possibility now exists to test and compare male and female trees for the purposes of bark-cloth or paper production, and to conduct breeding experiments, although the genetic diversity present in Hawaii is limited compared to that present in Asia. Sexual dimorphism in some dioecious plants is linked to economically important traits such as disease resistance, leaf quality [33], or fruit production [35] In papaya (*Carica* sp.), a molecular sex marker has been used to select female plants and avoid unnecessary cultivation of males [35]. The sex marker for paper mulberry was originally developed to select in favor of male plants because of an unspecified higher value in China [32].

Our results are consistent with the observation of only female-inflorescences of paper mulberry in herbarium collections from Rapanui, Pitcairn and Tahiti, and the presence of females and putative male plants from Hawaii. The earliest female specimens (Table 2) were collected during the beginning of the 19th century, but are few in number and provide a numerically small window to the past. Although our present survey does not cover all known locations of paper mulberry in the Pacific, we can now be more confident that the prehistoric spread of this crop was entirely through vegetative propagation by people and that few, if any, male clones were introduced prehistorically.

Historical accounts from the first contact expeditions include descriptions of clothing or plantations, starting as early as 1606, and mention either the presence of bark-cloth or of paper mulberry trees on islands as far apart as Pitcairn, Tahiti, Rapanui, the Austral Islands, New Zealand and many more [1, 42]. In the absence of seeds or pollen, the only reliable way to determine the past sex distribution of paper mulberry in the Pacific is to identify the sex of plants in contexts where modern introductions are unlikely to have been made before the time of sample collection, and where it is known that paper mulberry was present at the time of first contact with Europeans. Samples from Sulawesi, New Guinea are female and were collected in areas where bark cloth culture was still present at the time of the collection.

In a previous paper [14] we discussed that we did not know where the G to T transversion, in the ribosomal ITS1 sequence occurred and which is characteristic of the Remote Oceanian populations. We had suggested that the G to T transversion occurred somewhere between Taiwan and Fiji. We can now limit the area where this change occurred to locations between New Guinea and Fiji.

We can now discuss the previous reports of a general absence of paper mulberry in Borneo and the Philippines [3, 11, 43, 44]. It was suggested that paper mulberry underwent a genetic bottleneck in passing from mainland Southeast Asia through Indonesia to Remote Oceania. The lack of botanical reports of the tree in the Philippines and Borneo, despite intensive botanical surveys in both regions, indicated a general absence in both regions [43]. The present data permit a new interpretation that explains this apparent absence, despite the new genetic evidence for an origin of Oceanic paper mulberry in southern Taiwan [11]. If only a single sex was introduced prehistorically to the Philippines, and then subsequently to Borneo and Indonesia, it might have survived only in areas where bark-cloth traditions were preserved until recently. There is no climatic limitation on the potential of paper mulberry to establish breeding populations in island Southeast Asia, as demonstrated by historical reports of such populations becoming established and naturalizing locally after recent introduction to the Philippines [44] and Solomon Islands (ref. in [3]). Today, the plant may be extinct in Borneo and the Philippines, or it may be very rare because no breeding populations were established, and because bark-cloth culture was largely abandoned in favour of woven textiles. In the absence of active propagation and cultivation by farmers, paper mulberry clones may have become extinct for a variety of reasons, as noted previously in the case of New Zealand, where the tree was present at the time of early European contact but disappeared soon afterwards [3]. There is still a possibility that early introductions of the plant will be re-discovered in New Zealand, and this may be the case in the Philippines and Borneo. In New Zealand, the production of bark-cloth was already very limited by the late 18^th^ century, before European settlement, most likely because of an early cultural shift by Maori to the use of native flax (*Phormium tenax*), a naturally abundant fiber plant used for woven and plaited textiles.

In the Philippines and Borneo, ethnographic records of bark-cloth production could be used to guide a search for surviving clones of paper mulberry. If these can be found in contexts that are not obvious locations for modern introduction, we predict that they will be female with the ITS-1 variant G and the chloroplast haplotype cp-17 [11]. Although it is well known that other taxa are used for bark-cloth production in those regions (e.g. *Antiaris toxicaria* [44]), ethnographic records of bark-cloth production do not include identification of all the taxa that are used or potentially usable in each area. If relict clonal stands of *B*. *papyrifera* can be found in the Philippines or Borneo, they may also point to locations or areas where archaeological evidence for early bark-cloth production can be found. Of course, it is to be expected that the tree, if introduced to a particular location at an early date, through migration or exchange, would continue to be moved to other locations over various distances, and across cultural boundaries (e.g. between Austronesian-speaking and Non-Austronesian speaking peoples). There is not necessarily a direct physical correspondence between the locations of living clonal stands and locations of early Austronesian settlement. The approach suggested here is just one of many ways that can be used, in principle, to narrow the search area for archaeological evidence of early Austronesian activities (including bark-cloth production) in Southeast Asia [45, 46, 47, 48]. The converse is also true. Any location where evidence of early bark-cloth production is provided by the presence of stone beaters, or other archaeological evidence, would also be a good starting place, in principle, to look for nearby stands of paper mulberry.

At present, there is some direct archaeological evidence for the early introduction of *B*. *papyrifera* into Polynesia. Orliac [49, 50] identified paper mulberry wood from several archaeological charcoal assemblages (dating from 690–220 yr BP) from Rapanui. At the Akahanga rockshelter site on Rapanui, *B*. *papyrifera* made up 32% of the total identified oven charcoal (48]. Similarly, at the site of Rano Kao on Rapanui, *Broussonetia* was identified in levels dating to pre-European times (610 ± 80 yr BP) based on charcoal identification [49]. Flenley et al. [51] also identified *B*. *papyrifera* pollen collected from a sedimentary lake core deposit on Rapanui, although palynological identification of *B*. *papyrifera* is problematic, because of the morphological similarity with pollen from other species of the Moraceae and also the Urticaceae and a range of other introduced plants of economic importance [30]. Horrocks [52] identified Moraceae pollen remains in an archaeological context in New Zealand, and suggested that they could be of paper mulberry. However, as the author pointed out, pollen of paper mulberry is difficult to differentiate from that of the two indigenous Moraceae species (*Streblus* spp.) [52]. On Huahine in the Society Islands, an archaeological tapa beater was recovered by Y. Sinoto from the early site of Vaito'otia, and provides circumstantial evidence for production of bark-cloth made of Moraceae (breadfruit, paper mulberry or *Ficus prolixa*) [53]. Kahn et al. [54] identified leaf hairs and possible pollen remains of paper mulberry in sediments retrieved from oven structures in the Opunohu Valley of Moorea (French Polynesia), suggesting that parts of the processed paper mulberry trunks were used as a fuel for cooking. To our knowledge, no other secure archaeobotanical material of this species has been identified from other islands in Polynesia.

The present observation of only female plants in all extant populations in Remote Oceania does not necessarily correspond to the initial expansion of Austronesian speakers into this region, but it does indicate a genetic bottleneck and a close cultural link to the early bark-cloth traditions of island Southeast Asia (through Sulawesi) and Taiwan. In the absence of breeding populations, the spread (i.e. movement) of paper mulberry depends entirely on a continuous human cultural tradition of preserving, propagating and transporting the plant. If self-maintained living female clones of ancient origin cannot be found in the Philippines and Borneo, or at any location along any other possible route of introduction into the Pacific, then it would appear that long-term survival of paper mulberry at any given location is also dependent on human care.

Under cultivation, clones of paper mulberry may be thousands of years old, and one possible direction for future genetic research is the development of a molecular clock based on the accumulation of mutations in different branches (ramets) of a single clonal lineage (genet). This approach has been used to estimate an age of 12,000 years for a naturally occurring genet of whispering aspen, *Populus tremuloides* [55]. To develop this approach in paper mulberry, it will be first necessary to use a sufficient number of genetic tests to characterize individual clones, and thus recognize ramets of a single clone in separate locations. A key question for future research is to determine whether several or just one female clone were introduced into island Southeast Asia and Remote Oceania.

## Supporting Information

S1 Fig Sequence of the male marker region. Bold and italics: primers designed for this study; underlined: primers designed by Wang et al. (30).
(TIF)

S1 Table List of contemporary plant material used in the present study. (DOCX)

S2 Table List of herbarium samples used in the present study. (DOCX)

## References

[1] Anderson E (1952) Plants, man and life. Boston: Little Brown & Co 245 p.

[2] Kirch P (2000) On the Road of the Winds: An Archaeological History of the Pacific Islands before European Contact. Berkeley: University of California Press 446 p.

[3] Matthews P (1996) Ethnobotany, and the origins of *Broussonetia papyrifera* in Polynesia: an essay on tapa prehistory In: Davidson JM, Irwin G, Leach BF, Pawley A, Brown D, editors. Oceanic Culture History: Essays in Honour of Roger Green. New Zealand: New Zealand Journal of Archaeology Special Publication pp 117–132.

[4] Matthews PJ (2007) Plant Trails in Oceania In: Howe KR, ed. Vaka Moana: Voyages of the Ancestors: The Discovery and Settlement of the Pacific. Honolulu: University of Hawai'i Press pp. 94–95.

[5] Whistler W (2009) Plants of the Canoe People: An Ethnobotanical Voyage through Polynesia. Lawai, Kauaʻi, Hawaiʻi: National Tropical Botanical Garden 241 p.

[6] Wilmshurst JM, Hunt TL, Lipo CP, Anderson AJ (2011) High-precision radiocarbon dating shows recent and rapid initial human colonization of East Polynesia. Proc Natl Acad Sci USA 108(5): 1815–20. 10.1073/pnas.1015876108 21187404 PMC3033267

[7] Jacomb C, Holdaway RN, Allentoft ME, Bunce M, Oskam CL, et al (2014) High-precision dating and ancient DNA profiling of moa (Aves: Dinornithiformes) eggshell documents a complex feature at Wairau Bar and refines the chronology of New Zealand settlement by Polynesians. J Archaeol Sci 50: 24–30.

[8] Matisoo-Smith E (2015) Ancient DNA and the human settlement of the Pacific: a review. J Hum Evol 79: 93–104. 10.1016/j.jhevol.2014.10.017 25556846

[9] Whistler W, Elevitch CR (2006) Traditional Trees of Pacific Islands: Their Culture, Environment, and Use. Holualoa, Hawaii: Permanent Agriculture Resources 800 p.

[10] Seelenfreund D, Clarke AC, Oyanedel N, Piña R, Lobos S, et al (2010) Paper mulberry (*Broussonetia papyrifera*) as a commensal model for human mobility in Oceania: anthropological, botanical and genetic considerations. New Zealand J Bot 48: 1–17.

[11] Chang C-S, Liu H-L, Moncada X, Seelenfreund A, Seelenfreund D, Chung K-F (2015) A holistic picture of Austronesian migrations revealed by phylogeography of Pacific paper mulberry. Proc Natl Acad Sci USA 112 (44): 13537–13542. 10.1073/pnas.1503205112 26438853 PMC4640734

[12] Zerega NJ, Clement WL, Datwyler SL, Weiblen GD (2005) Biogeography and divergence times in the mulberry family (Moraceae). Molec Phylogenet Evol 37: 402–416. 16112884 10.1016/j.ympev.2005.07.004

[13] Barker C (2002) "*Broussonetia papyrifera*, Moraceae." Curtis's Magazine 19(1): 8–18.

[14] Seelenfreund D, Piña R, Ho K-Y, Lobos S, Moncada X, Seelenfreund A (2011) Molecular analysis of *Broussonetia papyrifera* (L.) Vent. (Magnoliophyta: Urticales) from the Pacific, based on ribosomal sequences of nuclear DNA. New Zealand J Bot 49: 413–420.

[15] Gonzalez-Lorca J, Rivera-Hutinel A, Moncada X, Lobos S, Seelenfreund D, Seelenfreund A (2015) Ancient and modern introduction of *Broussonetia papyrifera* ([L.] Vent.; Moraceae) into the Pacific: Genetic, geographical and historical evidence. New Zealand J Bot 53: 75–89.

[16] Lebot V (2002) La domestication des plantes en Océanie et les contraintes de la voie asexuée. Journal de la Société des Océanistes 114–115: 46–61.

[17] Yoshida S, Matthews PJ (2002), Eds. Vegeculture in Eastern Asia and Oceania. Osaka: The Japan Center for Area Studies 335 p.

[18] Zerega NJC, Ragone D, Motley TJ (2004) Complex origins of breadfruit (*Artocarpus altilis*, Moraceae): Implications for human migrations in Oceania. Am J Bot 91: 760–766. 10.3732/ajb.91.5.760 21653430

[19] McKey D, Elias M, Pujol B, Duputié A (2010) The evolutionary ecology of clonally propagated domesticated plants. New Phytologist 186: 318–332. 10.1111/j.1469-8137.2010.03210.x 20202131

[20] Rodríguez W (2000) Botánica, domesticación y fisiología del cultivo de ñame (*Dioscorea alata*). Agronomía Mesoamericana 11: 133–152.

[21] Florence J (1997) Flore de la Polynésie Française. Vol 1 Paris: Orstom éditions 394 p.

[22] Zizka G (1991) Flowering plants of Easter Island. Frankfurt am Main, Germany, Palmengarten 108 p.

[23] Dubois A, Lenne P, Nahoe E, Rauch M (2013) Plantas de Rapa Nui. Guía ilustrada de la Flora de Interés Ecológico y Patrimonial Umanga mo te Natura. Santiago: CONAF, ONF International 132 p.

[24] Ghersa CM, de la Fuente E, Suarez S, Leon RJC (2002) Woody species in the Rolling Pampa grasslands, Argentina. Agriculture, Ecosystems and Environment 88: 271–278.

[25] Malik RN, Husain SZ (2007) *Broussonetia papyrifera* (L.) L’Her. Ex Vent.: an environmental constraint on the Himalayan foothills vegetation, Pakistan J Bot 39: 1045–1053.

[26] Morgan C, Overholt WA (2013) Wildland Weeds: Paper Mulberry, *Broussonetia papyrifera* Document is ENY-702, one of a series of the Entomology and Nematology Department, Florida Cooperative Extension Service, Institute of Food and Agricultural Sciences, University of Florida. Publication date: March 2004. Reviewed May 2010. Revised June 2013 (accessed. 20 August 2015).

[27] Yang Y.-L. & Chen S.-H. (1998) An investigation of airborne pollen in Taipei City, Taiwan, 1993–1994. J Plant Res 111: 501–508.

[28] Singh AB, Pandit T, Dahiya P (2003) Changes in airborne pollen concentrations in Delhi, India. Grana, 42(3): 168–177.

[29] Abbas S, Katelaris CH, Singh AB, Raza SM, Khan MA, Rashid M, et al (2012) World Allergy Organization Study on Aerobiology for Creating First Pollen and Mold Calendar with clinical significance in Islamabad, Pakistan; A Project of World Allergy Organization and Pakistan. Allergy, Asthma & Clinical Immunology Centre of Islamabad. World Allergy Org J 5(9): 103–110. 10.1097/WOX.0b013e31826421c8 PMC3651178 23283209

[30] Prebble M (2008) No fruit on that beautiful shore: What plants were introduced to the subtropical Polynesian islands prior to European contact? In: Clark G, Leach F, and O'Connor S, eds. Islands of inquiry: Colonisation, seafaring and the archaeology of maritime landscapes. Canberra: The Australian National University, ANU E-Press. Terra Australis 29: 227–248.

[31] Bachtrog D, Mank JE, Peichel CL, Kirkpatrick M, Otto SP, Ashman T-L, et al (2014) Sex determination: Why so many ways of doing it? PLoS Biol 12(7): e1001899 10.1371/journal.pbio.1001899 24983465 PMC4077654

[32] Wang L, Dai C, Liu D, Liu Q (2012) Identification of a male-specific amplified fragment length polymorphism (AFLP) marker in *Broussonetia papyrifera*. African J Biotechnol 11(33): 8196–8201.

[33] Wang D-W, Li Y, Li Z-Q (2011) Identification of a Male-Specific Amplified Fragment Length. Polymorphism (AFLP) and a Sequence Characterized Amplified Region (SCAR) Marker in *Eucommia ulmoides* Oliv. Int J Mol Sci 12: 857–864. 10.3390/ijms12010857 21340018 PMC3039984

[34] Adhikari S, Saha S, Bandyopadhyay TK, Ghosh P (2014) Identification and Validation of a New Male Sex-Specific ISSR Marker in Pointed Gourd (*Trichosanthes dioica* Roxb.) The Scientific World Journal Article ID 216896, doi.org/10.1155/2014/21689. 10.1155/2014/216896 PMC4236900 25538949

[35] Deputy JC, Ming R, Ma H, Liu Z, Fitch MM, Wang M, et al (2002) Molecular markers for sex determination in papaya (*Carica papaya* L.) Theor Appl Genet 106: 107–111. 12582877 10.1007/s00122-002-0995-0

[36] Kafkas S, Khodaeiaminjan M, Güney M, Kafkas E (2015) Identification of sex-linked SNP markers using RAD sequencing suggests ZW/ZZ sex determination in *Pistacia vera* L. BMC Genomics 16 (98): 1–11. 25765114 10.1186/s12864-015-1326-6 PMC4336685

[37] Lodhi MA, Guang-Ning Y, Norman FW & Bruce IR (1994) A simple and efficient method for DNA extraction from grapevine cultivars and *Vitis* species. Plant Mol Biol Rep 12: 6–13.

[38] Moncada X, Payacán C, Arriaza F, Lobos S, Seelenfreund D, Seelenfreund A 2013 DNA Extraction and Amplification from Contemporary Polynesian Bark-Cloth. Plos One 8(2): e56549 10.1371/journal.pone.0056549 23437166 PMC3578839

[39] Blattner FR (1999) Direct amplification of the entire ITS region from poorly preserved plant material using recombinant PCR. BioTechniques 27: 1180–1186. 10631497 10.2144/99276st04

[40] Robert CE (2004) MUSCLE: Multiple sequence alignment with high accuracy and high throughput. Nucl Acids Res 32: 1792–1797. 15034147 10.1093/nar/gkh340 PMC390337

[41] Tamura K, Stecher G, Peterson D, Filipski A, Kumar S (2013) MEGA6: Molecular Evolutionary Genetics Analysis Version 6.0. Mol Biol Evol 30: 2725–2729. 10.1093/molbev/mst197 24132122 PMC3840312

[42] Merrill ED (1954) The botany of Cook’s voyages and its unexpected significance in relation to anthropology, biogeography, and history. Waltham, Mass., Chronica Botanica Co Pp 384 https://catalog.hathitrust.org/Record/002140266 (consulted online 09 July 2016).

[43] Primack RB, Ashton P (1987) Forester’s Guide to the Moraceae of Sarawak, East Malaysia In: Lugo AE et al, editors. People and the Tropical Forest. Washington DC: United States Man and the Biosphere Program pp. 45–47.

[44] Florece LM, Coladilla JO (2006) Spatial distribution and dominance of paper mulberry (*Broussonetia papyrifera*) in the vicinities of Mt. Makiling, Philippines. J Environ Science Management 9(2): 54–65.

[45] Howard M (2006) Bark-cloth of Southeast Asia Studies in the Material Cultures of Southeast Asia 10. Bangkok: White Lotus Press 327 p

[46] Sieveking G de G (1956) The Distribution of Stone Bark Cloth Beaters in Prehistoric Times. J Malayan Branch of the Royal Asiatic Society 29(3): 78–85.

[47] Ling SS (1962) Stone bark cloth beaters of South China, Southeast Asia and Central America. Paper presented at the Second Biennial Conference of Internatinal Association of Historian of Asia, Taipei, Taiwan available at www.ioe.sinica.edu.tw/…/FilesDownload.ashx? (Accessed 02.02.2016).

[48] Bellwood P (1993) Cultural and Biological Differentiation in Peninsular Malaysia: The Last 10,000 Years. Asian Perspectives 32 (1): 37–60.

[49] Orliac C (1998) Données nouvelles sur la composition de la flore de l'île de Pâques. Journal de la Société des Océanistes 107: 135–143.

[50] Orliac C (2000) The woody vegetation of Easter Island between the early 14th to the mid-17th centuries AD In: Stevenson CM, Ayres WS, editors. Easter Island Archaeology: Research on Early Rapanui culture Easter Island Foundation, Los Osos: Bearsville and Cloudmountain Presses, California pp. 199–207.

[51] Flenley JR, King SM, Jackson J, Chew C (1991) The Late Quaternary vegetational and climatic history of Easter Island. J Quat Science 6:85–115.

[52] Horrocks M, Smith IWG, Nichol SL, Wallace R (2008) Sediment, soil and plant microfossil analysis of Maori gardens at Anaura Bay, eastern North Island, New Zealand: comparison with descriptions made in 1769 by Captain Cook’s expedition. J Archaeol Sci 35: 2446–2464.

[53] Lepofsky D (2003) The Ethnobotany of Cultivated Plants of the Maohi of the Society Islands Econ Bot 57(1): 73–92.

[54] Kahn J, Horrocks M, Nieuwoudt N (2014) Agriculture, Domestic Production, and Site Function: Microfossil Analyses and Late Prehistoric Landscapes of the Society Islands. Econ Bot 68(3): 246–263.

[55] de Witte LC, Stöcklin J (2010) Longevity of clonal plants: why it matters and how to measure it. Ann Bot 106: 859–870. 10.1093/aob/mcq191 20880935 PMC2990663

