# Correction: Sex Distribution of Paper Mulberry (*Broussonetia papyrifera*) in the Pacific

**DOI:** 10.1371/journal.pone.0163188

**Published:** 2016-09-14

**Authors:** Johany Peñailillo, Gabriela Olivares, Ximena Moncada, Claudia Payacán, Chi-Shan Chang, Kuo-Fang Chung, Peter J. Matthews, Andrea Seelenfreund, Daniela Seelenfreund

A funding source has been omitted from the published article. The correct Financial Disclosure is: This project was funded by Fondecyt grants 1080061 and 1120175 from the Government of Chile to AS, and National Science Council, Taiwan Grant NSC-102-2621-B-002-007 to KFC. CSC received support from the Office of World Austronesian Studies, Bureau of International Cultural and Educational Affairs, Ministry of Education, Taiwan. The funders had no role in study design, data collection and analysis, decision to publish, or preparation of the manuscript.

There are omissions in the Author Contributions section of the published article. The correct information is: Conceived and designed the experiments: XM, JP; Contributed reagents/materials/analysis tools: AS, DS, CSC, KFC, PM; Performed the experiments: JP, GO, CP; Analyzed the data: JP, GO, CP, XM, DS; Wrote the manuscript: DS, AS, XM, PM.
