# Supplementary figures and images for: Sex Distribution of Paper Mulberry (Broussonetia papyrifera) in the Pacific
Source: PLoS One. 2016 Aug 16;11(8):e0161148. doi: 10.1371/journal.pone.0161148 (PMC4986985; doi:10.1371/journal.pone.0161148)

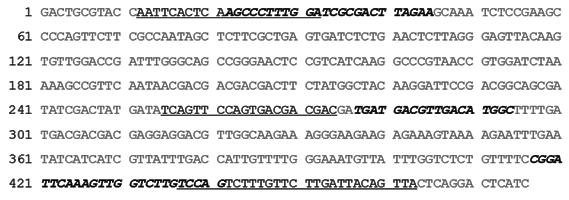

Supplement: S1 Fig — Bold and italics: primers designed for this study; underlined: primers designed by Wang et al. (30). (TIF) [file pone.0161148.s001.tif]
