# Supplementary material for: Sex Distribution of Paper Mulberry (Broussonetia papyrifera) in the Pacific
Source: PLoS One. 2016 Aug 16;11(8):e0161148. doi: 10.1371/journal.pone.0161148 (PMC4986985; doi:10.1371/journal.pone.0161148)
Supplement: S1 Table — (DOCX) [file pone.0161148.s002.docx]

| **.CONTEMPORARY LEAF SAMPLES** | | |  |  | |  | | | |
| --- | --- | --- | --- | --- | --- | --- | --- | --- | --- |
|  | **Location** | |  | **Genotype** | | **UTM Coordinates (datum WGS 84)** | | | |
| **Country** | **Archipelago/** | **Island/** | **Sample No.** | **ITS-1** | **Sex marker** | **Zone** | **East** | **North/ South** | **Hemi-sphere** |
|  | **Island/** | **Locality** |  |  |  |  |  |  |  |
|  | **Region** |  |  |  |  |  |  |  |  |
| Indonesia | Sulawesi | Tenagh/Pandere | CSC002 | G | F | 50 | 829.728 | 9.868.066 | S |
|  | Sulawesi | Tenagh/Pandere | CSC003 | G | F | 50 | 829728 | 9.868.066 | S |
|  | Sulawesi | Tenagh/Pandere | CSC1 | G | F | 50 | 829728 | 9.868.066 | S |
|  | Sulawesi | Tenagh/Bewa | CSC37 | G | F | 50 | 833709 | 9.887.341 | S |
|  | Sulawesi | Tenagh/Bewa | CSC38 | G | F | 50 | 833709 | 9.887.341 | S |
|  | Sulawesi | Tenagh/Gintu | CSC43 | G | F | 51 | 192773 | 9.790.611 | S |
|  | Sulawesi | Tenagh/Gintu | CSC44 | G | F | 51 | 192773 | 9.790.611 | S |
|  | Sulawesi | Tenagh/Lengkeka | CSC55-1 | G | F | 51 | 187747 | 9.797.622 | S |
|  | Sulawesi | Tenagh/Lengkeka | CSC55-2 | G | F | 51 | 187747 | 9.797.622 | S |
|  | Sulawesi | Tenagh/Lengkeka | CSC55-3 | G | F | 51 | 187747 | 9.797.622 | S |
|  | Sulawesi | Tenagh/Lengkeka | CSC56 | G | F | 51 | 187747 | 9.797.622 | S |
|  | Sulawesi | Tenagh/Gintu | CSC59-1 | G | F | 51 | 193859 | 9.791.057 | S |
|  | Sulawesi | Tenagh/Gintu | CSC59-2 | G | F | 51 | 193859 | 9.791.057 | S |
|  | Sulawesi | Tenagh/Gintu | CSC59-3 | G | F | 51 | 193859 | 9.791.057 | S |
|  | Sulawesi | Tenagh/Gintu | CSC59-4 | G | F | 51 | 193859 | 9.791.057 | S |
|  | Sulawesi | Tenagh/Gintu | CSC59-4 | G | F | 51 | 193859 | 9.791.057 | S |
| Solomon Islands | Guadalcanal | Honiara/ Kukum Hwy | Yang 23448 | G | F | 57 | 610874 | 8.957.773 | S |
|  | Guadalcanal | Honiara/ Kukum Hwy | Yang 23449 | G | M | 57 | 610874 | 8.957.773 | S |
| *New Caledonia | Grande Terre | Noumea, Museum of New Caledonia | BQUCH0442 | T | F | 58 | 648704 | 7535919 | S |
|  | Grande Terre | Noumea, Museum of New Caledonia | BQUCH0443 | T | F | 58 | 648704 | 7535919 | S |
|  | Grande Terre | Noumea, Museum of New Caledonia | BQUCH0444 | T | F | 58 | 648704 | 7535919 | S |
| Fiji | Viti Levu | Suva | BQUCH0114 | T | F | 60 | 652575 | 7992503 | S |
|  | Viti Levu | Votua | BQUCH0115 | T | F | 60 | 575025 | 7986616 | S |
|  | Viti Levu | Votua | BQUCH0116 | T | F | 60 | 575025 | 7986616 | S |
|  | Viti Levu | Votua | BQUCH0117 | T | F | 60 | 575029 | 7986675 | S |
|  | Taveuni | Bauma District, Waitabu Village | BQUCH0246 | T | F | 1 | 195179 | 8138902 | S |
|  | Taveuni | Bauma District, Waitabu Village | BQUCH0247 | T | F | 1 | 195179 | 8138902 | S |
|  | Taveuni | Bauma District, Waitabu Village | BQUCH0248 | T | F | 1 | 195179 | 8138902 | S |
|  | Taveuni | Bauma District, Waitabu Village | BQUCH0249 | T | F | 1 | 195179 | 8138902 | S |
|  | Taveuni | Bauma District, Waitabu Village | BQUCH0250 | T | F | 1 | 195179 | 8138902 | S |
|  | Taveuni | Bauma District, Waitabu Village | BQUCH0251 | T | F | 1 | 195179 | 8138902 | S |
|  | Taveuni | Bauma District, Waitabu Village | BQUCH0252 | T | F | 1 | 195179 | 8138902 | S |
|  | Taveuni | Bauma District, Waitabu Village | BQUCH0253 | T | F | 1 | 195179 | 8138902 | S |
|  | Vanua Levu | Natewa | BQUCH0258 | T | F | 60 | 791806 | 8163645 | S |
|  | Vanua Levu | Natewa | BQUCH0259 | T | F | 60 | 791806 | 8163645 | S |
|  | Vanua Levu | Natewa | BQUCH0260 | T | F | 60 | 791806 | 8163645 | S |
|  | Vanua Levu | Natewa | BQUCH0261 | T | F | 60 | 791806 | 8163645 | S |
|  | Vanua Levu | Natewa | BQUCH0262 | T | F | 60 | 791806 | 8163645 | S |
|  | Vanua Levu | Natewa | BQUCH0263 | T | F | 60 | 791806 | 8163645 | S |
|  | Vanua Levu | Natewa | BQUCH0264 | T | F | 60 | 791806 | 8163645 | S |
|  | Vanua Levu | Natewa | BQUCH0265 | T | F | 60 | 791806 | 8163645 | S |
|  | Vanua Levu | Natewa | BQUCH0266 | T | F | 60 | 791806 | 8163645 | S |
|  | Vanua Levu | Natewa | BQUCH0267 | T | F | 60 | 791806 | 8163645 | S |
|  | Vanua Levu | Buca | BQUCH0275 | T | F | 60 | 803208 | 8156920 | S |
|  | Vanua Levu | Buca | BQUCH0276 | T | F | 60 | 803208 | 8156920 | S |
|  | Vanua Levu | Buca | BQUCH0277 | T | F | 60 | 803208 | 8156920 | S |
|  | Vanua Levu | Loa | BQUCH0278 | T | F | 60 | 799782 | 8153604 | S |
|  | Vanua Levu | Loa | BQUCH0279 | T | F | 60 | 799782 | 8153604 | S |
|  | Vanua Levu | Loa | BQUCH0280 | T | F | 60 | 799782 | 8153604 | S |
|  | Vanua Levu | Loa | BQUCH0281 | T | F | 60 | 800945 | 8154255 | S |
|  | Vanua Levu | Loa | BQUCH0282 | T | F | 60 | 800945 | 8154255 | S |
|  | Vanua Levu | Buca | BQUCH0283 | T | F | 60 | 803208 | 8156920 | S |
|  | Vanua Levu | Buca | BQUCH0284 | T | F | 60 | 803208 | 8156920 | S |
|  | Vanua Levu | Naidi | BQUCH0285 | T | F | 60 | 752213 | 8141459 | S |
|  | Vanua Levu | Naidi | BQUCH0286 | T | F | 60 | 752213 | 8141459 | S |
|  | Vanua Levu | Naidi | BQUCH0287 | T | F | 60 | 752173 | 8141380 | S |
|  | Vanua Levu | Naidi | BQUCH0288 | T | F | 60 | 752173 | 8141380 | S |
|  | Vanua Levu | Nayavita | BQUCH0289 | T | F | 60 | 748659 | 8140820 | S |
|  | Vanua Levu | Votua | BQUCH0290 | T | F | 60 | 683369 | 8156325 | S |
|  | Taveuni | Vuna | BQUCH0291 | T | F | 60 | 808317 | 8121369 | S |
|  | Taveuni | Vuna | BQUCH0292 | T | F | 60 | 808317 | 8121369 | S |
|  | Taveuni | Qacavuio | BQUCH0293 | T | F | 60 | 818532 | 8139392 | S |
|  | Taveuni | Waiyevo | BQUCH0294 | T | F | 1 | 181870 | 8142296 | S |
|  | Taveuni | Lovonivonu | BQUCH0295 | T | F | 1 | 182128 | 8142135 | S |
|  | Taveuni | Lovonivonu | BQUCH0296 | T | F | 1 | 182128 | 8142135 | S |
|  | Taveuni | Somo Somo | BQUCH0297 | T | F | 1 | 183294 | 8143208 | S |
|  | Taveuni | Mua | BQUCH0298 | T | F | 1 | 189433 | 8150504 | S |
|  | Taveuni | Navaka Doa | BQUCH0299 | T | F | 1 | 196416 | 8143427 | S |
|  | Taveuni | Navaka Doa | BQUCH0300 | T | F | 1 | 196416 | 8143427 | S |
|  | Taveuni | Wunindawa | BQUCH0301 | T | F | 1 | 194350 | 8138227 | S |
|  | Taveuni | Bouma | BQUCH0302 | T | F | 1 | 193775 | 8137546 | S |
|  | Taveuni | Bouma | BQUCH0303 | T | F | 1 | 193775 | 8137546 | S |
|  | Vatulele | Ekuba | BQUCH0304 | T | F | 60 | 567955 | 7952166 | S |
|  | Vatulele | Ekuba | BQUCH0305 | T | F | 60 | 567955 | 7952166 | S |
|  | Vatulele | Ekuba | BQUCH0306 | T | F | 60 | 567955 | 7952166 | S |
|  | Vatulele | Ekuba | BQUCH0307 | T | F | 60 | 567908 | 7951874 | S |
|  | Vatulele | Ekuba | BQUCH0308 | T | F | 60 | 567811 | 7951600 | S |
|  | Vatulele | Ekuba | BQUCH0309 | T | F | 60 | 567811 | 7951600 | S |
|  | Vatulele | Ekuba | BQUCH0310 | T | F | 60 | 567811 | 7951600 | S |
|  | Vatulele | Ekuba | BQUCH0311 | T | F | 60 | 567811 | 7951600 | S |
|  | Vatulele | Ekuba | BQUCH0312 | T | F | 60 | 567811 | 7951600 | S |
|  | Vatulele | Ekuba | BQUCH0313 | T | F | 60 | 567811 | 7951600 | S |
|  | Vatulele | Ekuba | BQUCH0314 | T | F | 60 | 567811 | 7951600 | S |
|  | Vatulele | Ekuba | BQUCH0315 | T | F | 60 | 567811 | 7951600 | S |
|  | Vatulele | Ekuba | BQUCH0316 | T | F | 60 | 567811 | 7951600 | S |
|  | Vatulele | Ekuba | BQUCH0317 | T | F | 60 | 567255 | 7951374 | S |
|  | Vatulele | Ekuba | BQUCH0318 | T | F | 60 | 567255 | 7951374 | S |
|  | Vatulele | Ekuba | BQUCH0319 | T | F | 60 | 567811 | 7951600 | S |
|  | Vatulele | Ekuba | BQUCH0320 | T | F | 60 | 567204 | 7951400 | S |
|  | Vatulele | Ekuba | BQUCH0321 | T | F | 60 | 567204 | 7951400 | S |
|  | Vatulele | Ekuba | BQUCH0322 | T | F | 60 | 567200 | 7951496 | S |
|  | Viti Levu | Suva | BQUCH0323 | T | F | 60 | 651623 | 7993144 | S |
|  | Viti Levu | Suva | BQUCH0324 | T | F | 60 | 651623 | 7993144 | S |
|  | Viti Levu | Suva | BQUCH0325 | T | F | 60 | 651623 | 7993144 | S |
|  | Viti Levu | Korova-Suva | BQUCH0326 | T | F | 60 | 653524 | 7992480 | S |
|  | Viti Levu | Korova-Suva | BQUCH0327 | T | F | 60 | 653524 | 7992480 | S |
| France (overseas territory) | Wallis | Utu fua | BQUCH0268 | T | F | 1 | 587231 | 8524898 | S |
|  | Wallis | Utu fua | BQUCH0269 | T | F | 1 | 587536 | 8526671 | S |
|  | Wallis | Ha'afuasia | BQUCH0270 | T | F | 1 | 588428 | 8529404 | S |
|  | Wallis | Kolopopo | BQUCH0271 | T | F | 1 | 585001 | 8524659 | S |
|  | Wallis | Utu fua | BQUCH0272 | T | F | 1 | 587057 | 8524836 | S |
|  | Wallis | Utu fua | BQUCH0273 | T | F | 1 | 587057 | 8524836 | S |
|  | Wallis | Utu fua | BQUCH0328 | T | F | 1 | 587514 | 8526318 | S |
|  | Wallis | Utu fua | BQUCH0329 | T | F | 1 | 587792 | 8528591 | S |
|  | Wallis | Mata Utu | BQUCH0330 | T | F | 1 | 589704 | 8532168 | S |
|  | Wallis | Ha' afuasia | BQUCH0331 | T | F | 1 | 588428 | 8529404 | S |
|  | Wallis | Vaimalau | BQUCH0332 | T | F | 1 | 583051 | 8528034 | S |
|  | Wallis | Kolopopo | BQUCH0333 | T | F | 1 | 585001 | 8524659 | S |
| Tonga | Tongatapu | Fatai | BQUCH0095 | T | F | 1 | 678952 | 7661967 | S |
|  | Tongatapu | Fatai | BQUCH0096 | T | F | 1 | 678952 | 7661967 | S |
|  | Tongatapu | Teekiu | BQUCH0097 | T | F | 1 | 674537 | 7662944 | S |
|  | Tongatapu | Teekiu | BQUCH0098 | T | F | 1 | 674537 | 7662944 | S |
|  | Tongatapu | Foui | BQUCH0099 | T | F | 1 | 672658 | 7664354 | S |
|  | Tongatapu | Kanokupolu | BQUCH0100 | T | F | 1 | 673088 | 7668514 | S |
|  | Tongatapu | Kanokupolu | BQUCH0101 | T | F | 1 | 673088 | 7668514 | S |
|  | Tongatapu | Liahona | BQUCH0102 | T | F | 1 | 679102 | 7659056 | S |
|  | Tongatapu | Liahona | BQUCH0103 | T | F | 1 | 679102 | 7659056 | S |
|  | Tongatapu | Vaini | BQUCH0104 | T | F | 1 | 690042 | 7654751 | S |
|  | Tongatapu | Niutoua | BQUCH0105 | T | F | 1 | 703140 | 7660724 | S |
|  | Tongatapu | Niutoua | BQUCH0106 | T | F | 1 | 703140 | 7660724 | S |
|  | Tongatapu | Navutoka | BQUCH0107 | T | F | 1 | 698178 | 7663183 | S |
|  | Tongatapu | Malapo | BQUCH0108 | T | F | 1 | 692129 | 7654777 | S |
|  | Tongatapu | Malapo | BQUCH0109 | T | F | 1 | 691752 | 7654064 | S |
|  | Tongatapu | Pelehake | BQUCH0110 | T | F | 1 | 694042 | 7647088 | S |
|  | Tongatapu | Pelehake | BQUCH0111 | T | F | 1 | 694043 | 7647089 | S |
|  | Tongatapu | Hamula | BQUCH0112 | T | F | 1 | 695595 | 7649045 | S |
|  | Tongatapu | Fatumu | BQUCH0113 | T | F | 1 | 695999 | 7653459 | S |
|  | Tongatapu | Nukualofa | BQUCH0338 | T | F | 1 | 686924 | 7661960 | S |
|  | Tongatapu | Hofoa | BQUCH0339 | T | F | 1 | 684719 | 7663295 | S |
|  | Tongatapu | Halaleva | BQUCH0340 | T | F | 1 | 687682 | 7660132 | S |
|  | Tongatapu | Vila | BQUCH0341 | T | F | 1 | 683554 | 7658754 | S |
|  | Tongatapu | Alaki Fonua | BQUCH0342 | T | F | 1 | 693866 | 7655314 | S |
|  | Tongatapu | Tofoa | BQUCH0343 | T | F | 1 | 683611 | 7689544 | S |
|  | Tongatapu | Tofoa | BQUCH0344 | T | F | 1 | 683576 | 7659577 | S |
|  | Tongatapu | Havelo | BQUCH0345 | T | F | 1 | 684870 | 7660294 | S |
|  | Tongatapu | Hofoa | BQUCH0346 | T | F | 1 | 684147 | 7661604 | S |
|  | Tongatapu | Fatai | BQUCH0347 | T | F | 1 | 679926 | 7661952 | S |
|  | Tongatapu | Talasio | BQUCH0348 | T | F | 1 | 678636 | 7662108 | S |
|  | Tongatapu | Masilamea | BQUCH0349 | T | F | 1 | 674182 | 7663051 | S |
|  | Tongatapu | Ha'atafu | BQUCH0350 | T | F | 1 | 673931 | 7669432 | S |
|  | Tongatapu | Kanokupolu | BQUCH0351 | T | F | 1 | 672563 | 7667274 | S |
|  | Tongatapu | Fahefa | BQUCH0352 | T | F | 1 | 673573 | 7660581 | S |
|  | Tongatapu | Liahona | BQUCH0353 | T | F | 1 | 678130 | 7659666 | S |
|  | Tongatapu | Tokomololo | BQUCH0354 | T | F | 1 | 681782 | 7657557 | S |
|  | Vava'u | Neiafu | BQUCH0355 | T | F | 2 | 185222 | 7935049 | S |
|  | Vava'u | Neiafu | BQUCH0356 | T | F | 2 | 185245 | 7935048 | S |
|  | Vava'u | Holonga | BQUCH0357 | T | F | 2 | 188886 | 7943313 | S |
|  | Vava'u | Koloa | BQUCH0358 | T | F | 2 | 191865 | 7936036 | S |
|  | Vava'u | Tefisi | BQUCH0359 | T | F | 2 | 814838 | 7937263 | S |
|  | Vava'u | Utu-ngake | BQUCH0360 | T | F | 2 | 815337 | 7937469 | S |
|  | Eua | Fungafonua | BQUCH0361 | T | F | 1 | 713581 | 7632938 | S |
|  | Eua | Lakufaanga | BQUCH0362 | T | F | 1 | 714964 | 7627710 | S |
|  | Eua | Lakufaanga | BQUCH0363 | T | F | 1 | 714964 | 7627710 | S |
|  | Eua | Lakufaanga | BQUCH0364 | T | F | 1 | 716033 | 7626828 | S |
|  | Eua | Lakufaanga | BQUCH0365 | T | F | 1 | 716429 | 7626500 | S |
|  | Eua | Haaluma | BQUCH0366 | T | F | 1 | 713113 | 7629489 | S |
|  | Eua | Pangai | BQUCH0367 | T | F | 1 | 712086 | 7636237 | S |
|  | Eua | Houma | BQUCH0368 | T | F | 1 | 715059 | 7642374 | S |
|  | Eua | Taanga | BQUCH0369 | T | F | 1 | 712413 | 7639218 | S |
|  | Eua | Taanga | BQUCH0370 | T | F | 1 | 712413 | 7639218 | S |
| Samoa | Upolu | Siumu | BQUCH0067 | T | F | 2 | 415485 | 8453117 | S |
|  | Upolu | Siumu | BQUCH0068 | T | F | 2 | 415485 | 8453117 | S |
|  | Savaii | Salailua | BQUCH0069 | T | F | 2 | 326870 | 8484191 | S |
|  | Savaii | Palauli | BQUCH0074 | T | F | 2 | 357751 | 8479413 | S |
|  | Savaii | Safu'a | BQUCH0075 | T | F | 2 | 369464 | 8483379 | S |
|  | Savaii | Faga | BQUCH0077 | T | F | 2 | 371327 | 8490104 | S |
|  | Savaii | Siutu | BQUCH0082 | T | F | 2 | 326966 | 8483875 | S |
|  | Savaii | Siutu | BQUCH0083 | T | F | 2 | 326966 | 8483875 | S |
|  | Savaii | Salailua | BQUCH0084 | T | F | 2 | 326870 | 8484191 | S |
|  | Savaii | Salailua | BQUCH0085 | T | F | 2 | 326870 | 8484191 | S |
|  | Savaii | Palauli | BQUCH0086 | T | F | 2 | 357751 | 8479413 | S |
|  | Savaii | Palauli | BQUCH0087 | T | F | 2 | 357751 | 8479413 | S |
|  | Savaii | Safu'a | BQUCH0088 | T | F | 2 | 369464 | 8483379 | S |
|  | Savaii | Safu'a | BQUCH0089 | T | F | 2 | 369464 | 8483379 | S |
|  | Savaii | Faga | BQUCH0090 | T | F | 2 | 371327 | 8490104 | S |
|  | Savaii | Faga | BQUCH0091 | T | F | 2 | 371327 | 8490104 | S |
|  | Upolu | Maangiangi | BQUCH0092 | T | F | 2 | 371327 | 8490104 | S |
|  | Upolu | Maangiangi | BQUCH0093 | T | F | 2 | 415731 | 8450887 | S |
|  | Upolu | Apia | BQUCH0094 | T | F | 3 | 420121 | 8468643 | S |
| French Polynesia | Tahiti | Pirae | BQUCH0056 | T | F | 6 | 229136 | 8057689 | S |
|  | Tahiti | Punauia | BQUCH0058 | T | F | 6 | 222970 | 8048528 | S |
|  | Tahiti | Punauia | BQUCH0432 | T | F | 6 | 222970 | 8048528 | S |
|  | Tahiti | Punauia | BQUCH0433 | T | F | 6 | 222970 | 8048528 | S |
|  | Tahiti | Punauia | BQUCH0434 | T | F | 6 | 222970 | 8048528 | S |
|  | Tahiti | Papara | BQUCH0435 | T | F | 6 | 234119 | 8034576 | S |
|  | Raiatea | Opoa | BQUCH0063 | T | F | 5 | 673114 | 8137905 | S |
|  | Marquesas | Ua Pou, Haka Hau | BQUCH0040 | T | F | 7 | 604344 | 8964915 | S |
|  | Marquesas | Ua Pou, Haka Hetau | BQUCH0041 | T | F | 7 | 598300 | 8965159 | S |
|  | Marquesas | Nuku Hiva, Taoihae | BQUCH0042 | T | F | 7 | 597261 | 9018056 | S |
|  | Marquesas | Hiva Oa, Atu Ona | BQUCH0043 | T | F | 7 | 715199 | 8915659 | S |
|  | Marquesas | Fatuhiva, Omoa | BQUCH0045 | T | F | 7 | 753475 | 8836997 | S |
|  | Marquesas | Fatuhiva, Omoa | BQUCH0046 | T | F | 7 | 753475 | 8836997 | S |
|  | Marquesas | Fatuhiva, Hanavave | BQUCH0047 | T | F | 7 | 755608 | 8842390 | S |
|  | Marquesas | Fatuhiva, Hanavave | BQUCH0048 | T | F | 7 | 755608 | 8842390 | S |
|  | Marquesas | Tahuata, Vai Tahu | BQUCH0049 | T | F | 7 | 707417 | 8900911 | S |
|  | Marquesas | Tahuata, Vai Tahu | BQUCH0050 | T | F | 7 | 707417 | 8900911 | S |
|  | Marquesas | Tahuata, Hapatoni | BQUCH0051 | T | F | 7 | 705731 | 8897319 | S |
|  | Marquesas | Nukuhiva, Hatiheu | BQUCH0233 | T | F | 7 | 600999 | 9024025 | S |
|  | Marquesas | Fatuhiva, Omoa | BQUCH0234 | T | F | 7 | 753479 | 8836997 | S |
|  | Austral Islands (Rapa) | Ahurei | BQUCH0236 | T | F | 6 | 763082 | 6942048 | S |
|  | Austral Islands (Rapa) | Ahurei | BQUCH0439 | T | F | 6 | 763082 | 6942048 | S |
| British overseas territory | Pitcairn | Adamstown | BQUCH0060 | T | F | 9 | 389092 | 7227202 | S |
|  | Pitcairn | Adamstown | BQUCH0061 | T | F | 9 | 389092 | 7227202 | S |
|  | Pitcairn | Adamstown | BQUCH0062 | T | F | 9 | 389092 | 7227202 | S |
|  | Pitcairn | Adamstown | BQUCH0136 | T | F | 9 | 389092 | 7227202 | S |
|  | Pitcairn | Adamstown | BQUCH0440 | T | F | 9 | 389092 | 7227202 | S |
|  | Pitcairn | Adamstown | BQUCH0441 | T | F | 9 | 389092 | 7227202 | S |
| Chile | Rapa Nui | Roiho | BQUCH0001 | T | F | 12 | 658282 | 6999883 | S |
|  | Rapa Nui | Roiho | BQUCH0002 | T | F | 12 | 658282 | 6999883 | S |
|  | Rapa Nui | Roiho | BQUCH0003 | T | F | 12 | 658282 | 6999883 | S |
|  | Rapa Nui | Roiho | BQUCH0004 | T | F | 12 | 658282 | 6999883 | S |
|  | Rapa Nui | Roiho | BQUCH0005 | T | F | 12 | 658088 | 6999883 | S |
|  | Rapa Nui | Poike | BQUCH0006 | T | F | 12 | 671059 | 6999051 | S |
|  | Rapa Nui | Poike | BQUCH0007 | T | F | 12 | 671059 | 6999051 | S |
|  | Rapa Nui | Poike | BQUCH0009 | T | F | 12 | 671059 | 6999051 | S |
|  | Rapa Nui | Poike | BQUCH0010 | T | F | 12 | 671691 | 6999278 | S |
|  | Rapa Nui | Poike | BQUCH0011 | T | F | 12 | 671691 | 6999278 | S |
|  | Rapa Nui | Maunga Toatoa | BQUCH0012 | T | F | 12 | 668684 | 6997597 | S |
|  | Rapa Nui | Maunga Toatoa | BQUCH0013 | T | F | 12 | 668684 | 6997597 | S |
|  | Rapa Nui | Maunga Toatoa | BQUCH0014 | T | F | 12 | 668684 | 6997597 | S |
|  | Rapa Nui | Maunga Toatoa | BQUCH0015 | T | F | 12 | 668684 | 6997597 | S |
|  | Rapa Nui | Rano Raraku | BQUCH0023 | T | F | 12 | 670319 | 6998489 | S |
|  | Rapa Nui | Rano Raraku | BQUCH0024 | T | F | 12 | 670319 | 6998489 | S |
|  | Rapa Nui | Rano Kao | BQUCH0027 | T | F | 12 | 654421 | 6991509 | S |
|  | Rapa Nui | Rano Kao | BQUCH0028 | T | F | 12 | 654449 | 6991595 | S |
|  | Rapa Nui | Rano Kao | BQUCH0029 | T | F | 12 | 654588 | 6991542 | S |
|  | Rapa Nui | Rano Kao | BQUCH0030 | T | F | 12 | 654588 | 6991542 | S |
|  | Rapa Nui | Rano Kao | BQUCH0031 | T | F | 12 | 654620 | 6990505 | S |
|  | Rapa Nui | Rano Raraku | BQUCH0032 | T | F | 12 | 670269 | 6998457 | S |
|  | Rapa Nui | Rano Raraku | BQUCH0033 | T | F | 12 | 670319 | 6998489 | S |
|  | Rapa Nui | Mataveri | BQUCH0035 | T | F | 12 | 654483 | 6994651 | S |
|  | Rapa Nui | Ara Tataki Rereo | BQUCH0036 | T | F | 12 | 656512 | 6998933 | S |
|  | Rapa Nui | Hanga Oteo | BQUCH0037 | T | F | 12 | 662483 | 7005684 | S |
|  | Rapa Nui | Hanga Oteo | BQUCH0038 | T | F | 12 | 662483 | 7005685 | S |
|  | Rapa Nui | PuToki Toki | BQUCH0135 | T | F | 12 | 667502 | 7002011 | S |
|  | Rapa Nui | National Park Service Nursery | BQUCH0146 | T | F | 12 | 654642 | 6994398 | S |
|  | Rapa Nui | Te Karava/Poike | BQUCH0147 | T | F | 12 | 671059 | 6999051 | S |
|  | Rapa Nui | Te Karava/Poike | BQUCH0148 | T | F | 12 | 671059 | 6999051 | S |
|  | Rapa Nui | Roiho | BQUCH0149 | T | F | 12 | 658282 | 6999883 | S |
|  | Rapa Nui | Roiho | BQUCH0150 | T | F | 12 | 658282 | 6999883 | S |
|  | Rapa Nui | Roiho | BQUCH0151 | T | F | 12 | 658282 | 6999883 | S |
|  | Rapa Nui | Ana Te Pahu | BQUCH0152 | T | F | 12 | 658088 | 7001652 | S |
|  | Rapa Nui | Ana Te Pahu | BQUCH0153 | T | F | 12 | 658089 | 7001652 | S |
|  | Rapa Nui | Ana Te Pahu | BQUCH0154 | T | F | 12 | 658089 | 7001652 | S |
|  | Rapa Nui | Oroi | BQUCH0155 | T | F | 12 | 668684 | 6997597 | S |
|  | Rapa Nui | Oroi | BQUCH0156 | T | F | 12 | 668684 | 6997597 | S |
|  | Rapa Nui | Oroi | BQUCH0157 | T | F | 12 | 668684 | 6997597 | S |
|  | Rapa Nui | Oroi | BQUCH0158 | T | F | 12 | 668684 | 6997597 | S |
|  | Rapa Nui | Oroi | BQUCH0159 | T | F | 12 | 668684 | 6997597 | S |
|  | Rapa Nui | Oroi | BQUCH0160 | T | F | 12 | 668684 | 6997597 | S |
|  | Rapa Nui | PuToki Toki | BQUCH0208 | T | F | 12 | 667517 | 7002017 | S |
|  | Rapa Nui | PuToki Toki | BQUCH0209 | T | F | 12 | 667511 | 7002019 | S |
|  | Rapa Nui | Ahu Kihi Kihi Rau Mea | BQUCH0210 | T | F | 12 | 667511 | 7002019 | S |
|  | Rapa Nui | Ahu Kihi Kihi Rau Mea | BQUCH0211 | T | F | 12 | 656512 | 6998933 | S |
|  | Rapa Nui | MaungaToaToa | BQUCH0212 | T | F | 12 | 668684 | 6997597 | S |
|  | Rapa Nui | MaungaToaToa | BQUCH0213 | T | F | 12 | 668684 | 6997597 | S |
|  | Rapa Nui | Te Karava/Poike | BQUCH0214 | T | F | 12 | 671059 | 6999051 | S |
|  | Rapa Nui | Te Karava/Poike | BQUCH0215 | T | F | 12 | 671059 | 6999051 | S |
|  | Rapa Nui | Vai Tara Kai Ua | BQUCH0216 | T | F | 12 | 663657 | 7005502 | S |
|  | Rapa Nui | Vai Tara Kai Ua | BQUCH0217 | T | F | 12 | 663657 | 7005502 | S |
|  | Rapa Nui | Vai Tara Kai Ua | BQUCH 0218 | T | F | 12 | 663657 | 7005502 | S |
|  | Rapa Nui | National Park Service Nursery | BQUCH 0219 | T | F | 12 | 654649 | 6994386 | S |
|  | Rapa Nui | National Park Service Nursery | BQUCH0220 | T | F | 12 | 654649 | 6994386 | S |
|  | Rapa Nui | Rano Kau | BQUCH0221 | T | F | 12 | 654908 | 6992526 | S |
|  | Rapa Nui | Rano Kau | BQUCH0222 | T | F | 12 | 654908 | 6992526 | S |
|  | Rapa Nui | Rano Kau | BQUCH0223 | T | F | 12 | 654908 | 6992526 | S |
|  | Rapa Nui | Rano Kau | BQUCH0224 | T | F | 12 | 654910 | 6992532 | S |
|  | Rapa Nui | Hanga Oteo | BQUCH0225 | T | F | 12 | 661086 | 7006106 | S |
|  | Rapa Nui | Hanga Oteo | BQUCH0226 | T | F | 12 | 662498 | 7005729 | S |
| USA | Hawai | Big Island, Waimea | BQUCH0059 | T | F | 5 | 222236 | 2217080 | N |
|  | Hawai | Big Island, Waimea | BQUCH0064 | G | M | 5 | 222236 | 2217080 | N |
|  | Hawai | Big Island, Waimea | BQUCH0065 | T | F | 5 | 222236 | 2217080 | N |
|  | Hawai | Big Island, Waimea | BQUCH0066 | T | F | 5 | 222236 | 2217080 | N |
|  | Hawai | Oahu, B. Museum | BQUCH0161 | T | F | 4 | 616973. | 2359435 | N |
|  | Hawai | Oahu, B. Museum | BQUCH0162 | T | F | 4 | 616973. | 2359435 | N |
|  | Hawai | Oahu, B. Museum | BQUCH0163 | T | F | 4 | 616973. | 2359435 | N |
|  | Hawai | Oahu, B. Museum | BQUCH0164 | T | F | 4 | 616973. | 2359435 | N |
|  | Hawai | Oahu, Lyon's arboretum | BQUCH0165 | G | M | 4 | 624219 | 2359576 | N |
|  | Hawai | Oahu, Lyon's arboretum | BQUCH0166 | G | M | 4 | 624219 | 2359576 | N |
|  | Hawai | Oahu, Waimea Valley | BQUCH0167 | G | M | 4 | 597934 | 2392608 | N |
|  | Hawai | Oahu, Waimea Valley | BQUCH0168 | G | M | 4 | 597934 | 2392608 | N |
|  | Hawai | Oahu, Waimea Valley | BQUCH0169 | G | M | 4 | 597934 | 2392608 | N |
|  | Hawai | Oahu, Waimea Valley | BQUCH0170 | T | F | 4 | 597934 | 2392608 | N |
|  | Hawai | Oahu, Waimea Valley | BQUCH0171 | G | M | 4 | 597934 | 2392608 | N |
|  | Hawai | Oahu, Waimea Valley | BQUCH0172 | G | M | 4 | 597934 | 2392608 | N |
|  | Hawai | Oahu, Waimea Valley | BQUCH0173 | G | M | 4 | 597934 | 2392608 | N |
|  | Hawai | Oahu, Lyon's arboretum | BQUCH0174 | T | F | 4 | 624219 | 2359576 | N |
|  | Hawai | Big Island, Waimea | BQUCH0176 | G | M | 5 | 222236 | 2217080 | N |
|  | Hawai | Big Island, Waimea | BQUCH0177 | G | M | 5 | 222236 | 2217080 | N |
|  | Hawai | Big Island, Kohala, Lapakahi | BQUCH0178 | T | F | 5 | 197160 | 2233525 | N |
|  | Hawai | Big Island, Kohala, Lapakahi | BQUCH0179 | T | F | 5 | 197160 | 2233525 | N |
|  | Hawai | Big Island, Waimea | BQUCH0180 | T | F | 5 | 222236 | 2217080 | N |
|  | Hawai | Big Island, Waimea | BQUCH0181 | T | F | 5 | 222236 | 2217080 | N |
|  | Hawai | Big Island, Waimea | BQUCH0182 | T | F | 5 | 222236 | 2217080 | N |
|  | Hawai | Big Island, Kona | BQUCH0183 | G | M | 5 | 194368 | 2157724 | N |
|  | Hawai | Big Island, Kona | BQUCH0184 | T | F | 5 | 194368 | 2157724 | N |
|  | Hawai | Big Island, Kona | BQUCH0185 | G | M | 5 | 194368 | 2157724 | N |
|  | Hawai | Big Island, Kona | BQUCH0186 | G | M | 5 | 194368 | 2157724 | N |
|  | Hawai | Big Island, Kona | BQUCH0187 | T | F | 5 | 194368 | 2157724 | N |
|  | Hawai | Big Island, Kona | BQUCH0188 | T | F | 5 | 194368 | 2157724 | N |
|  | Hawai | Big Island, Kona | BQUCH0189 | G | M | 5 | 194368 | 2157724 | N |
|  | Hawai | Oahu, Waimea Valley | BQUCH0190 | T | F | 4 | 597940 | 2392647 | N |
|  | Hawai | Big Island, Kona | BQUCH0191 | G | M | 5 | 194368 | 2157724 | N |
|  | Hawai | Big Island, Kona | BQUCH0192 | G | M | 5 | 194368 | 2157724 | N |
|  | Hawai | Big Island | BQUCH0193 | T | F | 5 | 194368 | 2157724 | N |
|  | Hawai | Oahu, Makaha Valley | BQUCH0194 | G | M | 4 | 581493 | 2374443 | N |
|  | Hawai | Oahu, Makaha Valley | BQUCH0195 | T | F | 4 | 581493 | 2374443 | N |
| Taiwan | Taiwan | Da Han river coast | BQUCH0118 | G | F | 51 | 330589 | 2755464 | N |
|  | Taiwan | Da Han river coast | BQUCH0119 | G | M | 51 | 327900 | 2775841 | N |
|  | Taiwan | Da Du river coast | BQUCH0120 | G | M | 51 | 310605 | 2705543 | N |
|  | Taiwan | Da Du river interior | BQUCH0121 | G | F | 51 | 258771 | 2700750 | N |
|  | Taiwan | Jhuo Shuei interior | BQUCH0122 | G | F | 51 | 281849 | 2627375 | N |
|  | Taiwan | Jhuo Shuei coast | BQUCH0123 | G | M | 51 | 200035 | 2554847 | N |
|  | Taiwan | Zeng Wun interior | BQUCH0124 | G | F | 51 | 244441 | 2568090 | N |
|  | Taiwan | Zeng Wun coast | BQUCH0125 | G | M | 51 | 231917 | 2493088. | N |
|  | Taiwan | Lao Nong interior | BQUCH0126 | G | M | 51 | 238414 | 2521362 | N |
|  | Taiwan | Lao Nong coast | BQUCH0127 | G | F | 51 | 235151 | 2490226 | N |
|  | Taiwan | Lan Yang coast | BQUCH0128 | G | M | 51 | 379492 | 2733670 | N |
|  | Taiwan | Lan Yang interior | BQUCH0129 | G | M | 51 | 351074 | 2721142 | N |
|  | Taiwan | Hua Lian coast | BQUCH0130 | G | M | 51 | 343203 | 2596530 | N |
|  | Taiwan | Hua Lian interior | BQUCH0131 | G | M | 51 | 322000 | 2574890 | N |
|  | Taiwan | Bei Nan river interior | BQUCH0132 | G | F | 51 | 284418 | 2498427 | N |
|  | Taiwan | Bei Nan river coast | BQUCH0133 | G | M | 51 | 293353 | 2499445 | N |
|  | Taiwan | Taichung | BQUCH0138 | G | M | 51 | 262551 | 2664757 | N |
|  | Taiwan | Wulai District | BQUCH0139 | G | F | 51 | 355499 | 2749171 | N |
|  | Taiwan | Wulai District | BQUCH0140 | G | F | 51 | 353385 | 2749479 | N |
| Vietnam | North Vietnam | Sapa Town | BQUCH0201 | G | M | 48 | 380542 | 2470619 | N |
|  | North Vietnam | Hanoi | BQUCH0202 | G | F | 48 | 580968 | 2325060 | N |
|  | North Vietnam | Ba Vi Mountain | BQUCH0203 | G | M | 48 | 537948 | 2329519 | N |
|  | North Vietnam | Ba Vi Mountain | BQUCH0204 | G | M | 48 | 537948 | 2329519 | N |
|  | North Vietnam | Ba Vi Mountain | BQUCH0205 | G | F | 48 | 537948 | 2329519 | N |
| Japan | Honshu | Kyoto | BQUCH0141 | G | M | 53 | 571342 | 3876355 | N |
|  | Honshu | Kyoto | BQUCH0142 | G | F | 53 | 572337 | 3877424 | N |
|  | Honshu | Kyoto (Vic. Kamosama, Kiyamachi St) | BQUCH0143 | G | M | 53 | 570301 | 3874083 | N |
|  | Honshu | Kyoto (Botanic Garden) | BQUCH0144 | G | F | 53 | 569569 | 3878734 | N |
|  | Honshu | Kyoto (Yamashima) | BQUCH0145 | G | F | 53 | 574145 | 3870936 | N |
| China | Guangdong Province | Guitou Township, Shaoguan City, Ruyuan County , | BQUCH0428 | G | F | 49 | 744327 | 2761353 | N |
|  | Guangdong Province | Guitou Township, Shaoguan City, Ruyuan County , | BQUCH0429 | G | F | 49 | 744327 | 2761353 | N |
|  | Guangdong Province | Guitou Township, Shaoguan City, Ruyuan County , | BQUCH0430 | G | M | 49 | 744327 | 2761353 | N |
|  | Guangdong Province | Tsuiheng Village, Zhongshan City, | BQUCH0431 | G | M | 49 | 760759 | 248548 | N |

* Currently paper mulberry is quite abundant in certain areas of New Caledonia, however these plants have all been recently introduced by Wallisian migrants settling in New Caledonia, who needed the plants for its cultural importance and lack of local plants. Samples in this study were carefully chosen by a resident from plants that are not associated to Wallisian settlers and possibly represent the remains of ancient stands.
