# Supplementary material for: Sex Distribution of Paper Mulberry (Broussonetia papyrifera) in the Pacific
Source: PLoS One. 2016 Aug 16;11(8):e0161148. doi: 10.1371/journal.pone.0161148 (PMC4986985; doi:10.1371/journal.pone.0161148)
Supplement: S2 Table — (DOCX) [file pone.0161148.s003.docx]

**S2 Table. List of herbarium samples used in the present study.**

| **Location** | |  |  |  | **Genotype** | |
| --- | --- | --- | --- | --- | --- | --- |
| **Archipelago** | **Island/**  **Location** | **Insitution** | **Accession No.** | **Collector (year)** | **ITS-1** | **Sex marker** |
| New Guinea | New Guinea (Ekua) | B.P. Bishop Museum | 161323 | Blackwood, B. (1973) | T | F |
| New Guinea | New Guinea, Kaironk Valley | Auckland Institute & Museum Herbarium | 116673 | Bulmer, R.N.H. (1964) | G | F |
| New Guinea | New Guinea | Eastern highland | NA | Brass 3076 | NA | F |
| New Guinea | New Guinea | Finisterre/ upper Naho Valley Range/ near Moro Village | NA | Jermy 4345 | NA | F |
| New Guinea | New Guinea | Finisterre Range/ upper Naho Valley/ near Moro Village | NA | Pullen 6192 | NA | F |
| Solomon Islands | Guadalcanal (Honiara) | B.P. Bishop Museum | 416666 | Krauss, N.L.H. (1977) | G | F |
| Solomon Islands | Guadalcanal (Honiara) | B.P. Bishop Museum | 505999 | Krauss N.L.H. (n.d.) | G | F |
| Solomon Islands | Guadalcanal (Honiara Botanical Garden) | Auckland Institute & Museum Herbarium | 214298 | Gardener, R.O. (1993) | G | F |
| Fiji | East Coast Koro island | B. P. Bishop Museum | 161326 | Smith A.C. 1097  (1994) | T | F |
| Fiji | Vanua Levu, Cakaudrove district, Vanua Levu | B. P. Bishop Museum | 757984 | Koriveibau D. 15560 (1968) | T | F |
| Fiji | Namuka i Lau, | B. P. Bishop Museum | 161324 | Bryan Jr. E.H. 474 (1924) | T | F |
| Fiji | Vanua Levu, Cakaudrove district | B. P. Bishop Museum | 32928 | Koriveibau D. 15559 (1968) | T | F |

NA: No information available; nd: no date available
